# PDGFRα^+^/Integrin α2^+^ Fibroblasts Orchestrate Tumor Budding in Oral Squamous Cell Carcinoma via Mechano‐Metabolic Symbiosis: E‐Cadherin/Integrin α2β1 Adhesion and Mitochondrial Transfer

**DOI:** 10.1002/advs.76385

**Published:** 2026-06-30

**Authors:** Yufang Liu, Jiao Li, Juan Liu, Qi Dong, Haoyang Zhang, Yanjin Wang, Huibing Li, Ye Guan, Lei Cao, Manqing Zhang, Fangning Guo, Xue Liu, Zhen Yang, Mengmeng Lu, Hui Liu, Laiping Zhong, Tong Ji, Tingjiao Liu

**Affiliations:** ^1^ Department of Oral Pathology Shanghai Stomatological Hospital & School of Stomatology Fudan University Shanghai China; ^2^ Shanghai Key Laboratory of Craniomaxillofacial Development and Diseases Shanghai Stomatological Hospital & School of Stomatology Fudan University Shanghai China; ^3^ Department of Orthodontics Shanghai Stomatological Hospital & School of Stomatology Fudan University Shanghai China; ^4^ Department of Multidisciplinary Consultant Center Shanghai Stomatological Hospital & School of Stomatology Fudan University Shanghai China; ^5^ Intelligent Medicine Institute Shanghai Medical College Fudan University Shanghai China; ^6^ Department of Oral and Maxillofacial Surgery Shanghai Stomatological Hospital & School of Stomatology Fudan University Shanghai China; ^7^ Huashan Hospital & School of Stomatology Fudan University Shanghai China; ^8^ Zhongshan Hospital & School of Stomatology Fudan University Shanghai China

**Keywords:** cancer‐associated fibroblasts, heterotypic adhesion, mitochondrial transfer, oral cavity squamous cell carcinoma, platelet‐derived growth factor receptor alpha, tumor budding

## Abstract

Tumor budding (TB), defined as single tumor cells or small clusters (≤4 cells) at the invasive front, is a strong adverse prognostic feature in oral squamous cell carcinoma (OSCC). However, the stromal regulators and molecular mechanisms that drive TB remain unclear. Here, we developed a tumor‐budding organoid (TBO) model that faithfully recapitulates the OSCC tumor‐stroma interface and budding dynamics. By integrating this model with single‐cell RNA sequencing (scRNA‐seq), in situ RNA sequencing (isRNA‐seq), and functional perturbations, we identify platelet‐derived growth factor receptor alpha^+^/integrin α2^+^ (PDGFRα^+^/integrin α2^+^) cancer‐associated fibroblasts (CAFs) as the key stromal subset promoting OSCC budding. OSCC cells recruit PDGFRα^+^ CAFs through PDGFA/PDGFRα signaling. These CAFs engage OSCC cells through two complementary crosstalk pathways: (1) heterotypic E‐cadherin/integrin α2β1 adhesion that transmits biomechanical cues to activate YAP signaling and induce epithelial‐mesenchymal transition (EMT)‐like programs; and (2) tunneling nanotube (TNT)‐mediated mitochondrial transfer that enhances oxidative phosphorylation (OXPHOS) and bioenergetic supply in budding cells. Targeted inhibition of TNT‐mediated mitochondrial transfer markedly suppresses TB in TBO and xenograft models. Together, our results reveal a mechano‐metabolic symbiosis between PDGFRα^+^/integrin α2^+^ CAFs and OSCC cells that drives TB and provides actionable targets to block this aggressive metastatic precursor.

## Graphical abstract

PDGFA/PDGFRα signaling recruits CAFs to OSCC nests, and CAF‐OSCC contact drives tumor budding invasion. Mechanistically, heterotypic adhesion via E‐cadherin/integrin α2β1 activates YAP signaling in OSCC cells, inducing EMT. Concurrently, CAFs transfer mitochondria through TNTs, supplying ATP to fuel invasion.

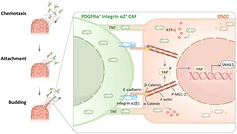

## 1 Introduction

Oral squamous cell carcinoma (OSCC), the predominant subtype of head and neck squamous cell carcinoma (HNSCC), accounts for ∼300 000 new cases and 145 000 deaths annually worldwide [1, 2]. A hallmark of OSCC aggressiveness is tumor budding (TB), a histopathological feature defined by detached single tumor cells or small clusters (≤4 cells) at the invasive front [3, 4, 5, 6, 7]. High‐grade TB is strongly associated with lymph node metastasis, therapy resistance, and reduced overall survival in OSCC [8, 9, 10]. Nevertheless, the cellular and molecular mechanisms that initiate and sustain TB remain poorly understood, in part because physiologically relevant models are lacking to capture dynamic tumor‐stroma interactions at the invasive front.

The tumor microenvironment (TME) is a major determinant of OSCC progression. Cancer‐associated fibroblasts (CAFs) are the most abundant and functionally diverse stromal cell type within the TME [11, 12]. CAFs can promote tumorigenesis through paracrine signaling, extracellular matrix (ECM) remodeling, and direct cell‐cell interactions [11, 13, 14, 15]. Although CAFs are enriched in TB regions of OSCC [7], direct evidence that specific CAF subsets initiate and sustain TB is limited. Moreover, CAFs exhibit profound functional heterogeneity [16, 17, 18], and the CAF subtypes and signaling programs that drive TB remain incompletely defined. This knowledge gap has hindered the development of targeted strategies to inhibit TB.

Traditional in vitro models (e.g., 2D monolayers and tumor spheroids) fail to reproduce the 3D architecture and contact‐dependent interactions at the tumor‐stroma interface [19, 20, 21]. Organoid technology has emerged as a powerful tool for modeling complex tissue dynamics, but most OSCC organoid platforms lack stromal components and cannot recapitulate TB. To address this limitation, we developed a TBO model that mimics the 3D cancer‐stroma interface and enables real‐time visualization of TB. Using this model, we combined high‐resolution transcriptomics (single‐cell RNA sequencing and in situ RNA sequencing) with functional assays to identify the CAF subset driving OSCC budding and to elucidate the underlying mechano‐metabolic crosstalk. Our findings reveal PDGFRα^+^/integrin α2^+^ CAFs as central regulators of TB through two coordinated mechanisms: biomechanical signaling via E‐cadherin/integrin α2β1 adhesion and bioenergetic support via TNT‐mediated mitochondrial transfer, providing a therapeutic framework for targeting this aggressive phenotype.

## 2 Results

### 2.1 Development of a Physiologically Relevant TBO Model Recapitulating OSCC Budding

To model OSCC nests surrounded by stromal CAFs in a controlled 3D setting, we fabricated a polydimethylsiloxane (PDMS) stamp‐based microcavity array within a type I collagen and Matrigel matrix (Figure 1A). Each stamp contained 36 microcavities (200 µm × 50 µm × 150 µm), enabling parallel culture of 72 organoids per 35 mm dish (Figure 1B). OSCC cells seeded into microcavities formed compact multicellular nests, whereas CAFs embedded in the surrounding matrix recapitulated the stromal TME (Figure 1C). OSCC nests maintained high viability (∼94%) (Figure 1D) and displayed robust TB at the cancer‐stroma interface, characterized by detachment of single cells or small clusters from the OSCC nest (Figure 1E). Time‐lapse imaging revealed a stereotyped budding sequence: CAFs migrated toward OSCC nests, established physical contact, and then retracted while maintaining adhesion to tumor cells, thereby pulling them outward to initiate budding (Figure 1F; Video S1). We observed two budding patterns, collective budding (CAFs at the leading edge) and independent budding (tumor cells alone), with collective budding predominating (Figure 1G,H).

**FIGURE 1:**
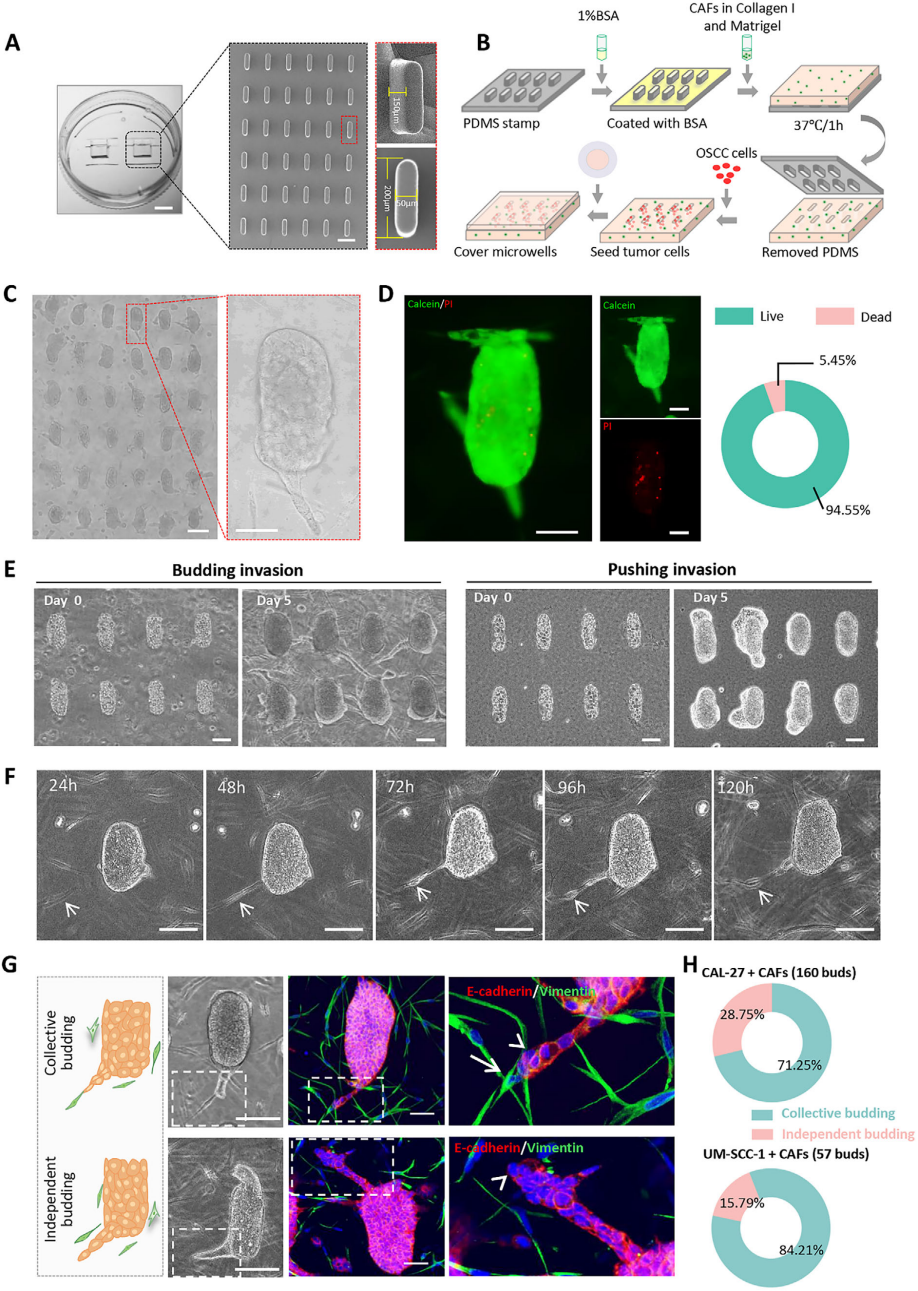
Construction of tumor organoids modeling OSCC budding invasion. (A) The PDMS stamp shown in a macroscopic photograph (left; scale bar = 5 mm) and the corresponding SEM image of its microstructured surface (right; scale bar = 200 µm). (B) Schematics of the establishment of the TBO models. (C) Bright‐field images showing 36 organoids within the fabricated 3D matrix (scale bar = 200 µm) and the corresponding magnified views (scale bar = 50 µm). (D) Live/Dead staining of CAL‐27 cells co‐cultured with CAFs in the TBO models, with quantitative analyses (Scale bars = 50 µm). (E) Representative images showing OSCC budding and pushing invasion pattern in the TBO and TPO models (Scale bars = 100 µm). (F) Representative images showing the trajectory of CAFs when promoting OSCC budding (Scale bars = 100 µm). (G) Representative images showing collective and independent budding of OSCC cells (brightfield images, Scale bars = 100 µm), (fluorescent images, Scale bars = 50 µm). (H) Quantitative analysis of collective and independent budding of OSCC cells (with 5 arrays for quantification). Data represent the mean (± SD); *n* = 5 per group.

As a control, we established a tumor pushing‐invasion organoid (TPO) model by culturing OSCC nests in a cell‐free matrix. In this setting, nests exhibited a pushing invasion pattern without discrete budding (Figure S1A). Addition of CAF‐conditioned medium to TPO cultures did not induce TB (Figure S1B), indicating that direct CAF‐OSCC contact, rather than soluble factors alone, is required to initiate budding. These data establish the TBO model as a physiologically relevant platform for studying contact‐dependent, CAF‐driven TB.

### 2.2 Combined scRNA‐seq and Spatial isRNA‐seq Characterize TB‐Promoting CAF Subtypes

To elucidate the cellular heterogeneity underlying TB, we performed scRNA‐seq on TBO and TPO models (18 060 high‐quality cells) and validated key spatial patterns by isRNA‐seq (Figure 2A). Unsupervised clustering identified 12 distinct cell clusters, including six CAF subpopulations (CAF‐C1 to CAF‐C6) and five OSCC subpopulations (OSCC‐C1 to OSCC‐C5) (Figure 2B,C,G).

**FIGURE 2:**
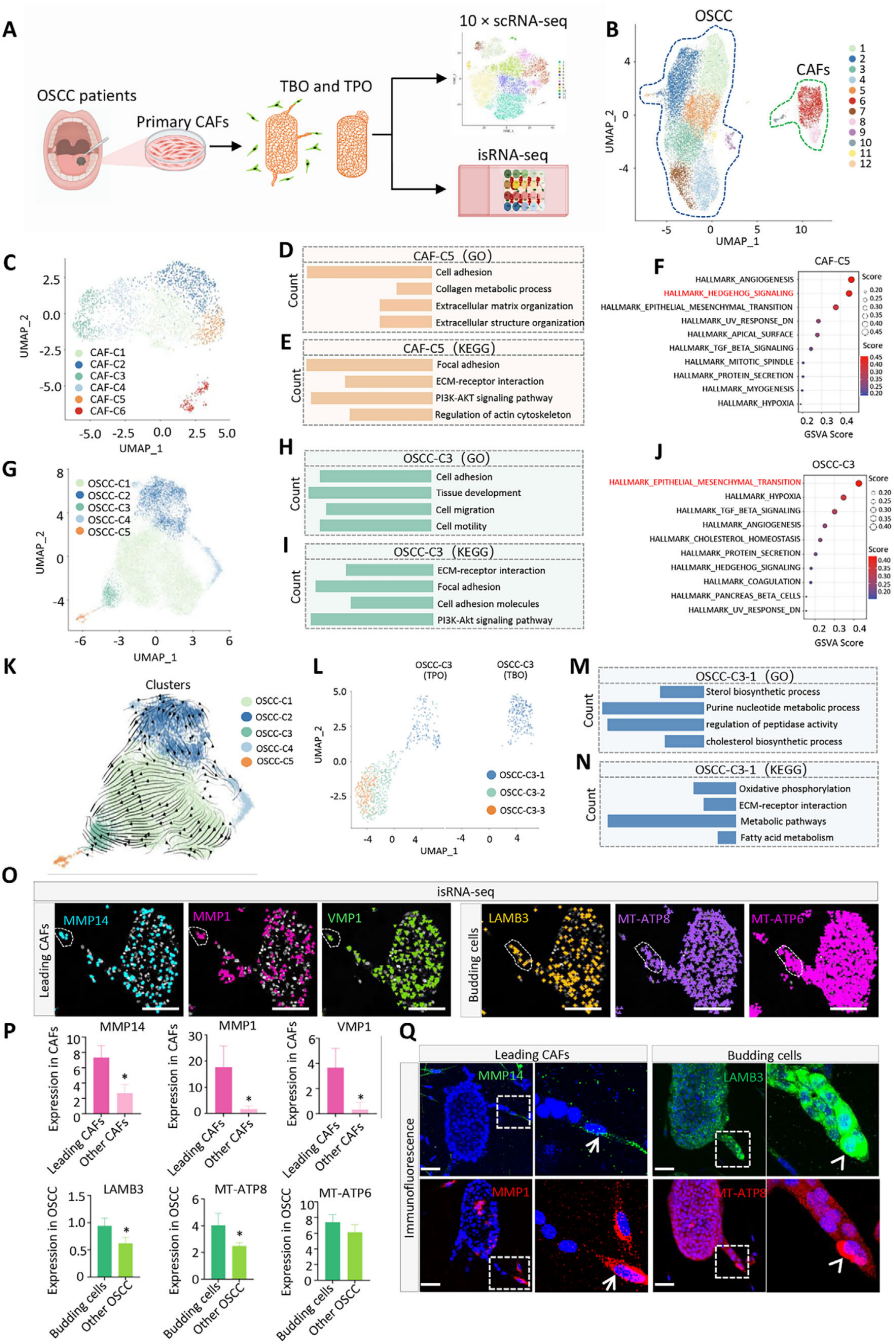
Characterization of leading CAFs and budding OSCC in the TBO models. (A) Schematic representation of the comprehensive experimental workflow combining scRNA‐seq with isRNA‐seq. (B) Analysis of scRNA‐seq data from both TBO and TPO models by UMAP, showing 18 060 single cells categorized into thirteen cell clusters. (C) UMAP plots displaying the re‐clustering of CAFs based on the scRNA‐seq. (D) Bar graph showing significantly enriched selected GO terms in CAF‐C5. (E) Bar graph showing significantly enriched selected KEGG terms in CAF‐C5. (F) Gene set variation analysis of CAF‐C5. (G) UMAP plots displaying the re‐clustering of OSCC based on the scRNA‐seq. (H) Bar graph showing significantly enriched selected GO terms in OSCC‐C3. (I) Bar graph showing significantly enriched selected KEGG terms in OSCC‐C3. (J) Gene set variation analysis of the OSCC‐C3 subtype. (K) Trajectory analysis of OSCC subtypes. Arrows indicate the extrapolated direction of development. (L) UMAP plots displaying the re‐clustering of OSCC‐C3 based on the scRNA‐seq. (M) Bar graph showing significantly enriched selected GO terms in OSCC‐C3‐1. (N) Bar graph showing significantly enriched selected KEGG terms in OSCC‐C3‐1. (O) Results of isRNA‐seq of the TBO model; the expression patterns of the following genes are color‐coded: LAMB3 – yellow, MT‐ATP8 – purple, MT‐ATP6 – magenta, MMP14 – cyan, MMP1 – plum red, and VMP1 – green (Scale bar = 100 µm). (P) Quantitative analyses of the expression levels of LAMB3, MT‐ATP8, MT‐ATP6, MMP14, MMP1, and VMP1, Data represent the mean (± SD); *n* = 3 per group. ^*^ *p* < 0.05 by *t*‐tests. (Q) The expression of LAMB3, MT‐ATP8, MMP14, and MMP1 proteins in the TBO models. Arrows indicated the leading CAFs, and arrowheads pointed to OSCC buds (Scale bars = 50 µm).

CAF‐C5 was uniquely enriched in gene ontology (GO) terms related to cell adhesion, ECM organization, and pro‐tumorigenic pathways (angiogenesis, Hedgehog signaling, EMT) (Figure 2D–F; Figure S2A–C), implicating this subset in TB. In contrast, other CAF subsets exhibited distinct functional phenotypes, including CAF‐C1 (inflammatory‐myofibroblastic), CAF‐C2 (inflammatory), CAF‐C3/C4 (proliferative), and CAF‐C6 (metabolic) (Figure S3). Among OSCC subsets, OSCC‐C3 displayed a strong invasive signature with enrichment in migration, EMT, and hypoxia pathways (Figure 2H–J; Figures S4A–C and S5). RNA velocity analysis confirmed OSCC‐C3 as a terminal invasive phenotype (Figure 2K). Further subclustering of OSCC‐C3 identified OSCC‐C3‐1 as a TBO‐specific subset (Figure 2L; Figure S4D) characterized by high expression of mitochondrial respiratory chain genes (MT‐ATP6, MT‐ND2) and enrichment in oxidative phosphorylation (OXPHOS) pathways (Figure 2M,N; Figures S4E–S6A–F), consistent with a distinct metabolic state linked to TB.

isRNA‐seq (39 probes) confirmed that CAF‐C5 marker genes (MMP14, MMP1, VMP1) were highly expressed in leading CAFs, whereas OSCC‐C3‐1 markers (LAMB3, MT‐ATP8, MT‐ATP6) were enriched in TB regions (Figure 2O,P; Figure S7A,B). Immunofluorescence (IF) staining validated these expression patterns (Figure 2Q), supporting CAF‐C5 as the leading CAF subset and OSCC‐C3‐1 as the budding OSCC subset.

### 2.3 PDGFRα^+^ CAFs are Recruited by OSCC Cells via PDGFA/PDGFRα Signaling to Promote TB

Time‐lapse imaging of TBO models revealed directional CAF migration toward OSCC nests, suggesting chemotactic recruitment. scRNA‐seq showed that PDGFA was selectively expressed in OSCC cells, whereas PDGFRα was enriched in CAFs, particularly within CAF‐C5 (Figure 3A,B). Western blotting confirmed PDGFA protein expression in OSCC cell lines (CAL‐27, UM‐SCC‐1) and PDGFRα upregulation in CAFs (Figure 3C). isRNA‐seq further demonstrated elevated PDGFRα levels in leading CAFs compared to non‐leading CAFs (Figure 3D).

**FIGURE 3:**
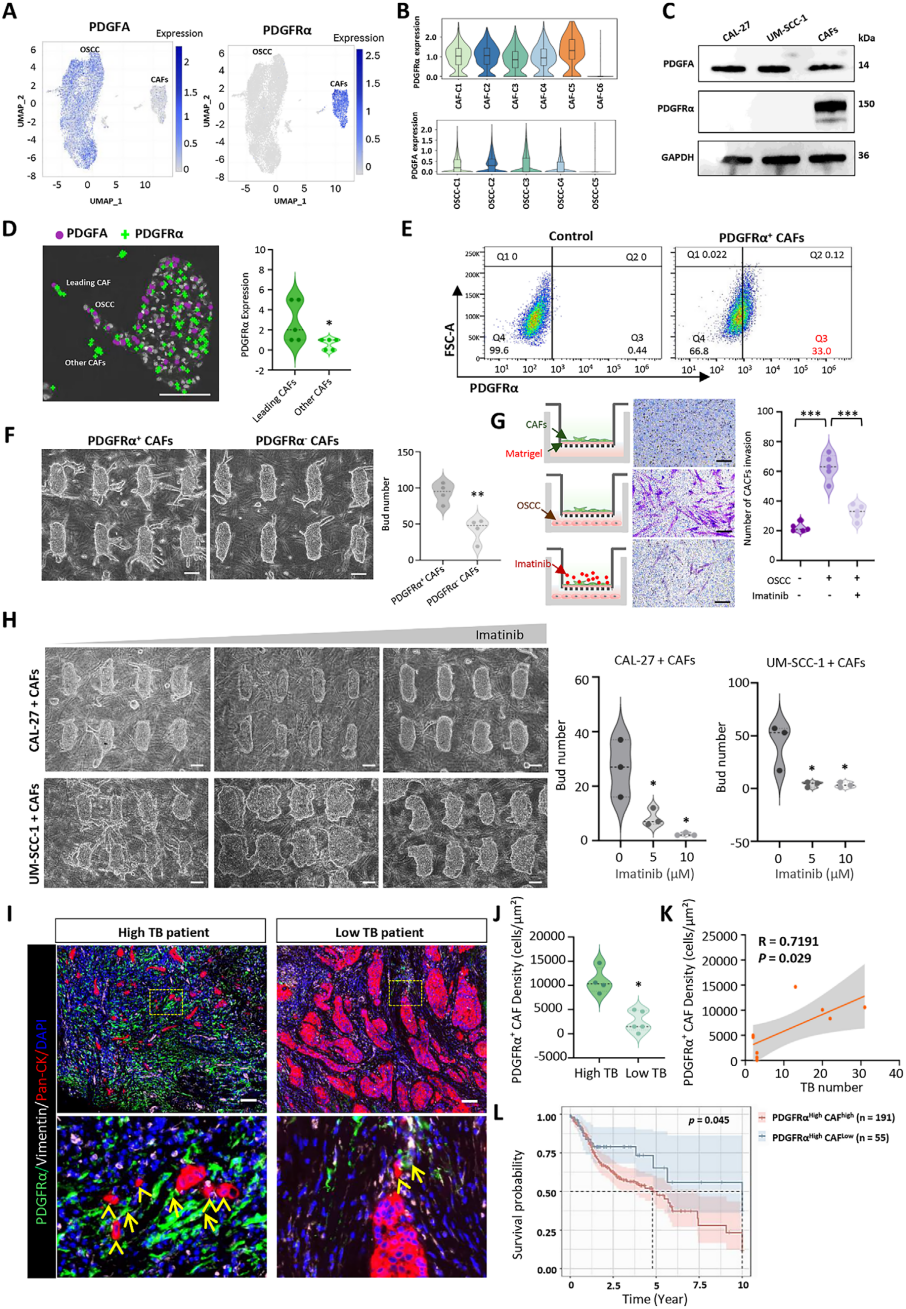
PDGFRα^+^ CAFs facilitate OSCC budding. (A,B) UMAP (A) and violin plots (B) showing the expression of PDGFA and PDGFRα in different subpopulations of CAFs and OSCC, respectively. (C) Immunoblot showing expression levels of PDGFA and PDGFRα in OSCC cell lines and CAFs. (D) Quantification of PDGFRα expression in the leading‐ and other CAFs using isRNA‐seq data. Data represent the mean (± SD); *n* = 5 per group. ^*^ *p* < 0.05 by t‐tests. Scale bar = 100 µm. (E) Flow cytometric analysis and sorting of PDGFRα^+^ CAFs and PDGFRα^−^ CAFs. (F) PDGFRα^+^ CAFs and PDGFRα^−^ CAFs were respectively co‐cultured with OSCC cells in the TBO models (Scale bars = 100 µm). The number of tumor buds in the PDGFRα^+^ and PDGFRα^−^ groups were compared. Data represent the mean (± SD); *n* = 4 per group. ^**^ *p* < 0.01 by t‐tests. (G) Representative images and quantification of the CAF invasion with or without OSCC stimulation and inhibitors (imatinib) (Scale bars = 100 µm). Data represent the mean (± SD); *n* = 5 per group. *n* = 5 per group. ^***^ *p* < 0.001 by One‐way ANOVA. (H) Imatinib inhibited CAF‐promoted OSCC budding in the TBO models in a concentration‐dependent manner (Scale bars = 100 µm). The numbers of tumor buds formed at different concentrations was compared. Data represent the mean (± SD); *n* = 3 per group. ^*^ *p* < 0.05, ^**^ *p* < 0.01 by One‐way ANOVA. (I,J) Analysis of PDGFRα^+^ CAFs (yellow arrows) and OSCC buds (yellow arrowheads) based on fluorescent staining, encompassing their spatial relationship, statistical comparison of PDGFRα^+^ CAF abundance between high TB and low TB cases (Scale bars = 100 µm). (K) Correlation analysis of the density of PDGFRα^+^ CAFs with TB number in patients. (L) Kaplan‐Meier curves showing the survival of HNSCC patients with high or low infiltration of PDGFRα^+^ CAFs in TCGA database (*n* = 246). The median expression of PDGFRα was used as the cutoff value.

Functional validation showed that flow cytometry‐sorted PDGFRα^+^ CAFs induced significantly more TB than PDGFRα^−^ CAFs in TBO models (Figure 3E,F). Transwell assays confirmed that OSCC cells recruit CAFs via PDGFA/PDGFRα signaling (Figure 3G; Figure S8A), and treatment with the PDGFRα inhibitor imatinib suppressed CAF‐induced TB (Figure 3H). Clinically, multiplex IF staining of human OSCC tissues showed that PDGFRα^+^ CAFs colocalized with budding cells and were more abundant in high‐TB cases (Figure 3I; Figure S8B). The abundance of PDGFRα^+^ CAFs differed significantly between high‐ and low‐TB specimens and showed a strong positive correlation with the number of TB (Figure 3J,K). TCGA analysis revealed that OSCC patients with high PDGFRα expression, together with a CAF signature, had worse overall survival (Figure 3L). Collectively, these data establish PDGFRα^+^ CAFs as critical mediators of TB recruited by OSCC cells through PDGFA/PDGFRα signaling.

### 2.4 Heterotypic E‐Cadherin/Integrin α2β1 Adhesion Transmits Mechanical Cues to Activate YAP Signaling

To identify cell‐cell interaction pathways between CAF‐C5 and OSCC‐C3‐1, we performed ligand‐receptor analysis using CellPhoneDB. The top‐ranked interaction was the CDH1‐ITGA2/ITGB1 complex (E‐cadherin/integrin α2β1) (Figure 4A). scRNA‐seq and isRNA‐seq confirmed compartmentalized expression: CDH1 (E‐cadherin) in OSCC cells and ITGA2/ITGB1 (integrin α2/β1) in CAFs (Figure 4B,C). Multicolor IF showed E‐cadherin enrichment in budding OSCC cells juxtaposed with integrin α2^+^/β1^+^ leading CAFs (Figure 4D; Figure S9A), confirming heterotypic E‐cadherin/integrin α2β1 adhesion. Functionally, treatment with TC‐I 15, a specific integrin α2β1 inhibitor, markedly suppressed CAF‐led tumor budding of OSCC cells in the TBO model (Figure 4E).

**FIGURE 4:**
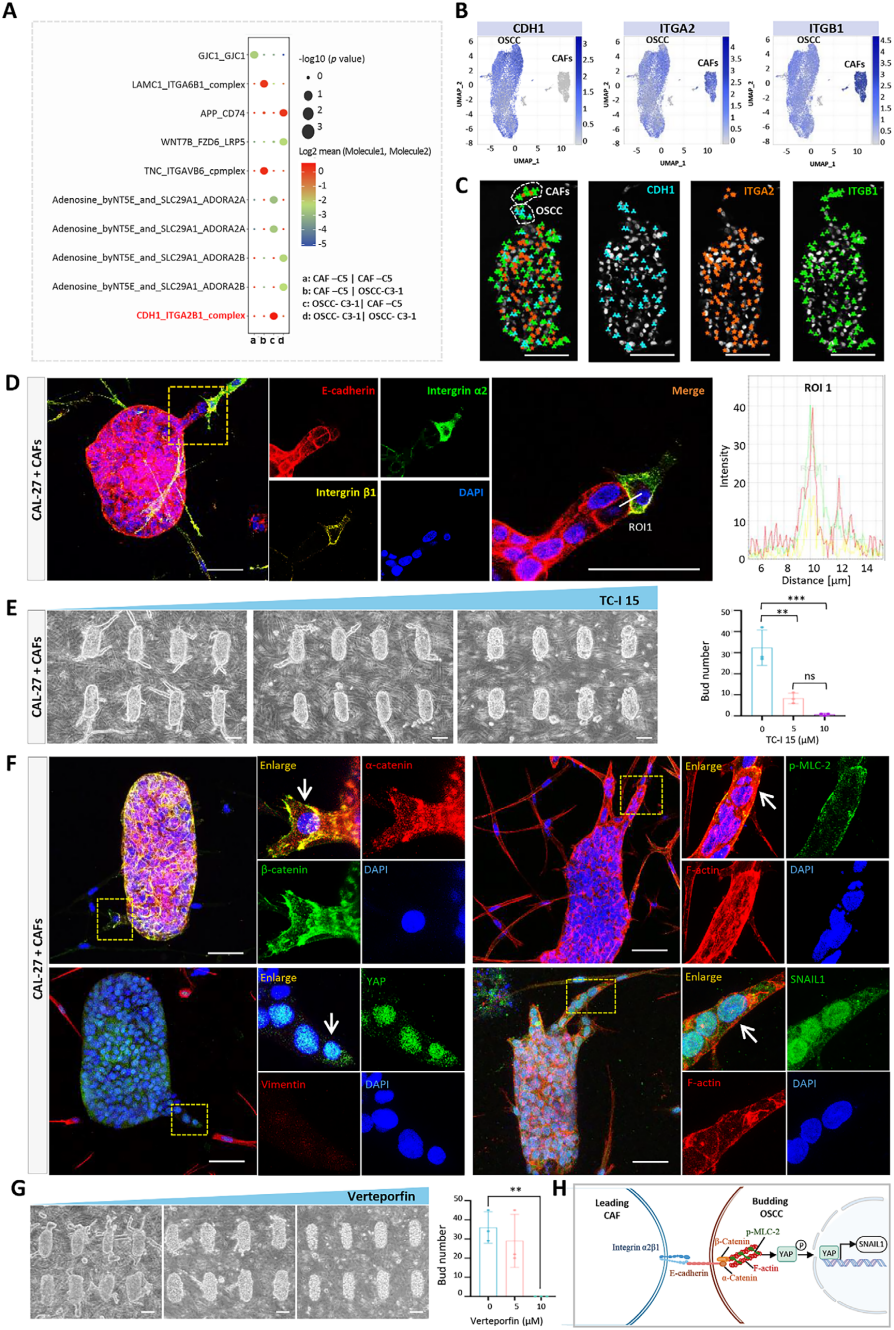
Leading CAFs and budding OSCC formed heterotypic E‐cadherin/integrin α2β1 adhesion and activated YAP signaling pathway in the OSCC cells. (A) The top 10 ligand‐receptor interactions between OSCC‐C3‐1 and CAF‐C5 clusters. (B) The expression of CDH1, ITGA2, and ITGB1 genes in the CAFs and OSCC based on scRNA‐seq data. (C) The expression of CDH1 (cyan), ITGA2 (orange), and ITGB1 (green) in the TBO models based on isRNA‐seq data (Scale bars = 100 µm). (D) Representative fluorescence images showing co‐localization of E‐cadherin (OSCC) with integrin α2β1 (CAFs) in the TBO models (Scale bars = 50 µm). (E) TC‐I 15 inhibited CAF‐promoted OSCC budding in the TBO models in a concentration‐dependent manner (Scale bars = 100 µm). Quantification of tumor buds in the different groups. Data represent the mean (± SD); *n* = 3 per group. ^**^ *p* < 0.01, ^***^ *p* < 0.001 by One‐way ANOVA. (F) Representative fluorescence images showing reorganization of the cytoskeleton and activation of YAP signaling pathway in the OSCC cells in the TBO models (Scale bars = 50 µm). (G) Verteporfin inhibited CAF‐promoted OSCC budding in the TBO models in a concentration‐dependent manner (Scale bars = 100 µm). Quantification of tumor buds in the different groups. Data represent the mean (± SD); *n* = 3 per group. ^**^ *p* < 0.01 by One‐way ANOVA. (H) Schematic representation of the interaction between leading CAFs and budding OSCC cells and the mechanism of YAP activation in OSCC.

Given the role of YAP as a key mediator of mechanical signaling, we examined YAP activation in budding cells. IF staining revealed cytoskeletal remodeling, including α‐catenin/β‐catenin colocalization and elevated p‐MLC‐2 (a marker of actomyosin contractility) in budding cells (Figure 4F; Figure S8B). Nuclear YAP localization and expression of the EMT regulator SNAIL‐1 were also observed in budding cells (Figure 4F; Figure S9B). Treatment with the YAP inhibitor verteporfin significantly suppressed CAF‐induced TB (Figure 4G). These data indicate that PDGFRα^+^ CAFs transmit mechanical cues via E‐cadherin/integrin α2β1 adhesion, activating YAP signaling and inducing EMT‐like phenotypes in OSCC cells to promote budding (Figure 4H).

### 2.5 TNT‐Mediated Mitochondrial Transfer Provides Bioenergetic Support for TB

OSCC‐C3‐1 cells exhibited high expression of mitochondrial genes and OXPHOS pathway enrichment (Figure 5A–C), suggesting increased bioenergetic demand. Transmission electron microscopy (TEM) showed that OSCC cells had smaller mitochondria with structural defects, whereas CAFs contained larger, intact mitochondria (Figure 5D). CAFs produced more ATP and had higher basal respiration than OSCC cells (Figure S10A–C). Coculture experiments revealed TNT formation between CAFs and OSCC cells (Figure 5E), and CAFs transferred functional mitochondria to OSCC cells via TNTs (Figure 5F; Figure S10D–F). Approximately 0.65%–1.02% of OSCC cells acquired CAF‐derived mitochondria (Figure 5G; Figure S10G). Metabolic profiling showed that OSCC cells receiving mitochondria had enhanced respiratory capacity and ATP production (Figure 5H,I).

**FIGURE 5:**
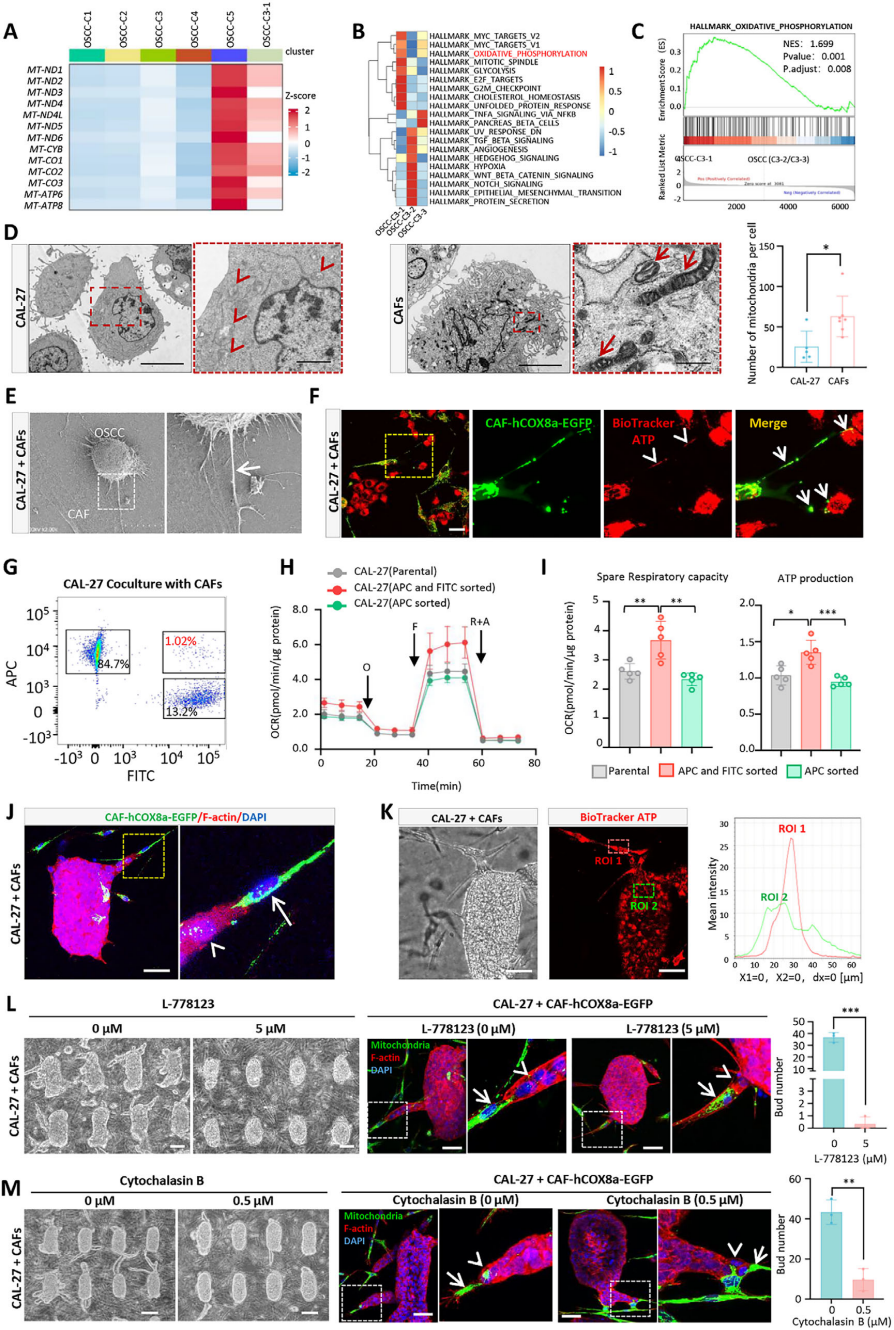
PDGFRα^+^/integrin α2^+^ CAFs transfer mitochondria to OSCC cells via TNTs. (A) Z‐score heatmap of mitochondria‐related gene expression in OSCC subpopulations based on scRNA‐seq data. (B) Gene set variation analysis of OSCC‐C3 subtypes. (C) Gene set enrichment analysis of OSCC‐C3‐1 vs. OSCC‐C3‐2 and OSCC‐C3‐3 in HALLMARK_ OXIDATIVE_PHOSPHORYLATION gene set. NES, normalized enrichment score. Exact *p*‐values are indicated. (D) Representative electron microscopy images of OSCC cells and CAFs (scale bars = 20 µm). Boxed regions are shown at higher magnification in the adjacent panels to depict mitochondrial morphology (scale bars = 500 nm), with representative examples indicated by red arrows. Corresponding quantitative analysis of mitochondrial number. Data are presented as the mean ± SD; ^*^ *p* ≤ 0.05 by Student's t‐test. (E) Representative SEM images showing the formation of TNTs between CAFs and OSCC cells. Arrow indicated a TNT (Scale bars = 20 µm). (F) Representative confocal micrographs of hCOX8a‐transfected CAFs co‐cultured with OSCC cells for 24 h and stained with BioTracker ATP Red. White arrows indicated mitochondria, and short white arrows pointed to the ATP signal produced by these mitochondria (Scale bars = 20 µm). (G) Flow cytometry analysis of E‐cadherin^+^ (APC) hCOX8a^+^ (FITC) cells after 48‐h co‐culture of hCOX8a‐transfected CAFs with OSCC cells. This population represents OSCC cells that have acquired mitochondria from CAFs. (H) The Oxygen Consumption Rate (OCR) was recorded in CAL‐27 cells under basal conditions and following the sequential injection of oligomycin (O), FCCP (F), and a mix of rotenone & antimycin A (R+A). Arrows indicated the time points of injections. Three experimental groups were analyzed: i) untreated parental CAL‐27 cells; ii) E‐cadherin‐positive (APC+) CAL‐27 cells sorted after 48 h of co‐culture with hCOX8a‐EGFP‐CAFs; and iii) CAL‐27 cells that had received CAF‐derived mitochondria, sorted as double‐positive for APC and FITC (APC+FITC+). (I) The spare respiratory capacity and ATP production were significantly increased in the recipient CAL‐27 cells. Data are presented as mean ± SD (*n* = 5 technical replicates per group). Statistical significance was determined by One‐way ANOVA (^*^ *p* ≤ 0.05, ^**^ *p* < 0.01, ^***^ *p* < 0.001). (J) Representative images of hCOX8a‐EGFP‐CAFs co‐cultured with OSCC cells in the TBO models showing CAFs delivering mitochondria (green) to OSCC cells. Arrow indicated a CAF and the arrow head indicated an OSCC cell (Scale bars = 50 µm). (K) Representative brightfield and BioTracker ATP staining images of the TBO models (Scale bars = 50 µm). The mean intensity of ROI1 is greater than ROI2. (L) Representative bright‐field/immunofluorescent images of CAF‐promoted OSCC budding with or without L‐778123 (scale bars = 100 µm) and quantification of tumor buds. Data are mean ± SD (*n* = 3 per group); ^***^ *p* < 0.001 by Student's t‐test. (M) Effects of different concentrations of cytochalasin B on OSCC budding in TBO models (scale bars, 100 µm) and quantitative analysis. Data are mean ± SD (*n* = 3 per group). Statistical significance was determined by Student's t‐test (^**^ *p* < 0.01).

In TBO models, mitochondrial transfer was confirmed in budding regions (Figure 5J; Figure S10H), and budding cells exhibited higher ATP levels than non‐budding cells (Figure 5K; Figure S10I). Treatment with the TNT‐inhibitor L‐778123, a farnesyl‐ and geranylgeranyltransferase inhibitor that indirectly disrupts TNT formation, blocked mitochondrial transfer and suppressed tumor budding (TB) (Figure 5L; Figure S10J,K). To directly target TNTs, we used cytochalasin B, an actin polymerization inhibitor that impairs TNT formation. Treatment of TBO cultures with 0.5 µm cytochalasin B markedly reduced tumor budding (Figure 5M). These data indicate that CAFs provide bioenergetic support for TB via TNT‐mediated mitochondrial transfer.

### 2.6 Inhibition of TNT‐Mediated Mitochondrial Transfer Suppresses TB in Xenograft Models

To validate in vivo relevance, we established OSCC xenografts by co‐injecting OSCC cells with CAFs derived from low‐ or high‐TB patients and treated mice with L‐778123 or PBS (Figure 6A). High‐TB patient‐derived CAFs (CAF‐L4) induced more TB than low‐TB CAFs (CAF‐L2) (Figure 6B–D; Figure S11A). Multiplex IF confirmed that PDGFRα^+^ CAFs colocalized with budding cells in xenografts (Figure S11B). L‐778123 treatment reduced TB in CAF‐L4 xenografts (Figure 6D) and in TBO models (Figure 6E,F), supporting the therapeutic potential of targeting TNT‐mediated mitochondrial transfer.

**FIGURE 6:**
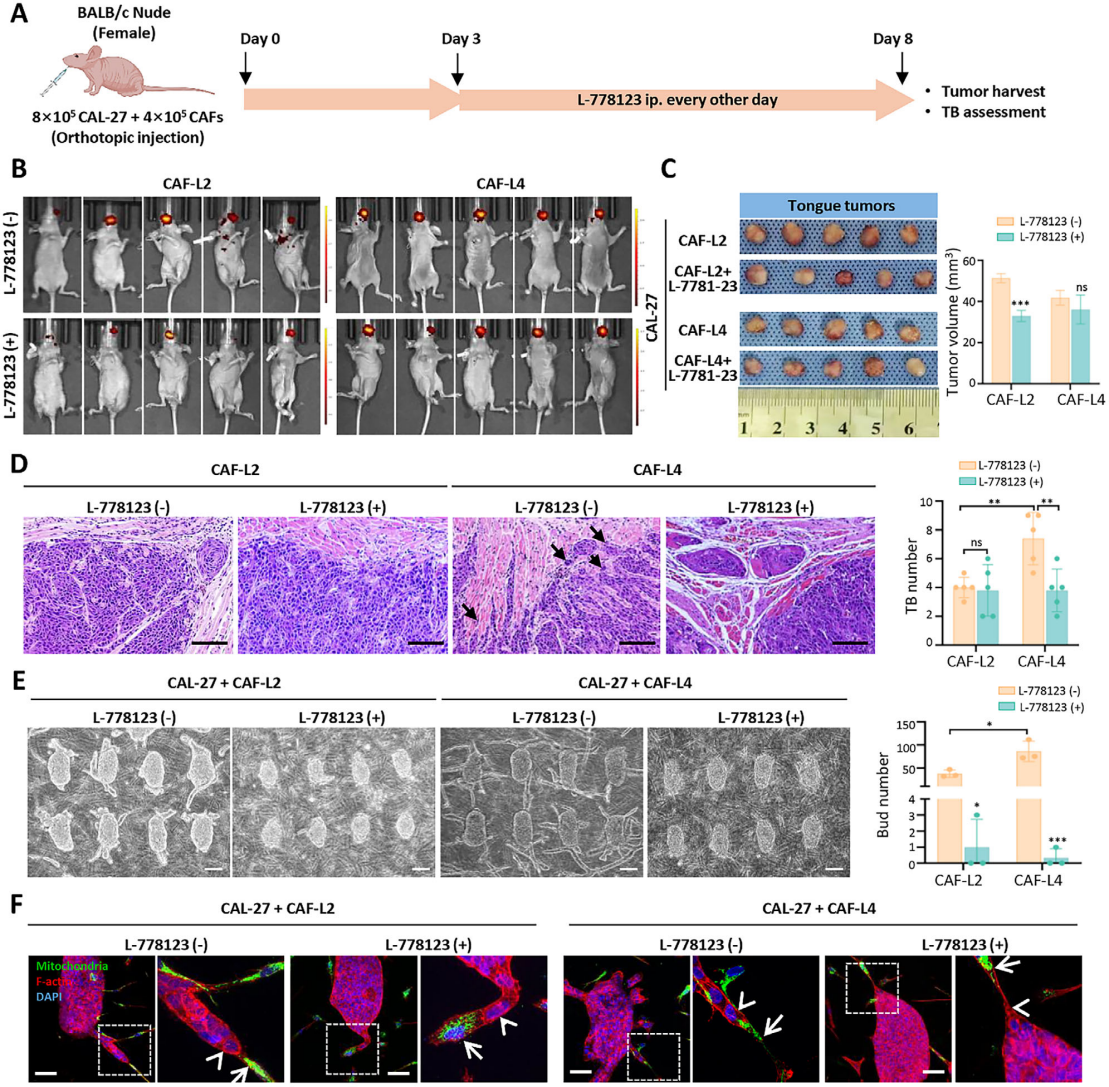
Inhibition of mitochondrial transfer from CAFs to OSCC can inhibit TB. (A) Schematic representation of establishing orthotopic xenografts using BALB/c nude mice. (B) In vivo imaging revealed orthotopic tumor development with or without L‐778123 on day 8. (C) Representative gross images of tumors and quantitative analyses of tumor volume. (D) HE‐stained tissue sections of xenografts (Scale bars = 100 µm). Quantitative analyses of tumor buds in the CAF‐L2, CAF‐L2 + L‐778123, CAF‐L4, and CAF‐L4 + L‐778123 groups (*n* = 5). ns, not significant and ^**^ *p* < 0.01 by Two‐way ANOVA. (E) CAF‐L2 and CAF‐L4 were co‐cultured with CAL‐27 with or without L‐778123 in the TBO models (Scale bars = 100 µm). Quantitative analyses of tumor buds in the indicated groups. Data represent the mean (± SD); *n* = 3 per group. ^*^ *p* < 0.05, ^***^ *p* < 0.001 by Two‐way ANOVA. (F) Representative fluorescence images of hCOX8a‐EGFP‐CAFs delivering mitochondria (green) to OSCC cells in the presence and absence of L‐778123 (Scale bars = 50 µm).

### 2.7 PDGFRα^+^/Integrin α2^+^ CAFs Are the Key TB‐Promoting Subset

To functionally evaluate the pro‐invasive capacity of CAFs derived from OSCC patients with distinct TB grades, we isolated CAFs from a cohort of 13 patients stratified by TB grade (low, *n* = 6; intermediate, *n* = 3; high, *n* = 4) and established TBO models for each (Figure 7A,B; Figure S12A,B). CAFs from high‐TB patients induced significantly more buds than those from low‐TB patients, while the intermediate group showed no statistical difference vs. others (Figure 7C). Correlation analysis revealed a significant positive association between the bud number in TBO models and the TB number in patient specimens (Figure 7D). Flow cytometry revealed that the proportion of PDGFRα^+^/integrin α2^+^ double‐positive CAFs was significantly elevated in high‐TB patients compared to low‐TB patients (Figure 7E; Figure S13D). In contrast, single‐positive populations (PDGFRα^+^, integrin α2^+^, or integrin β1^+^) were comparable across all TB groups (Figure S13A–C). Correlation analysis showed that the percentages of PDGFRα^+^ CAFs, integrin α2^+^ CAFs, and integrin β1^+^ CAFs were not significantly correlated with TB number (Figure S13E). By contrast, the percentage of PDGFRα^+^/integrin α2^+^ CAFs exhibited a significant positive correlation with both TB number and the bud number in TBO models (Figure S14A). Functionally, TBOs containing sorted PDGFRα^+^/integrin α2^+^ CAFs exhibited significantly more buds than those with double‐negative CAFs (Figure 7F).

**FIGURE 7:**
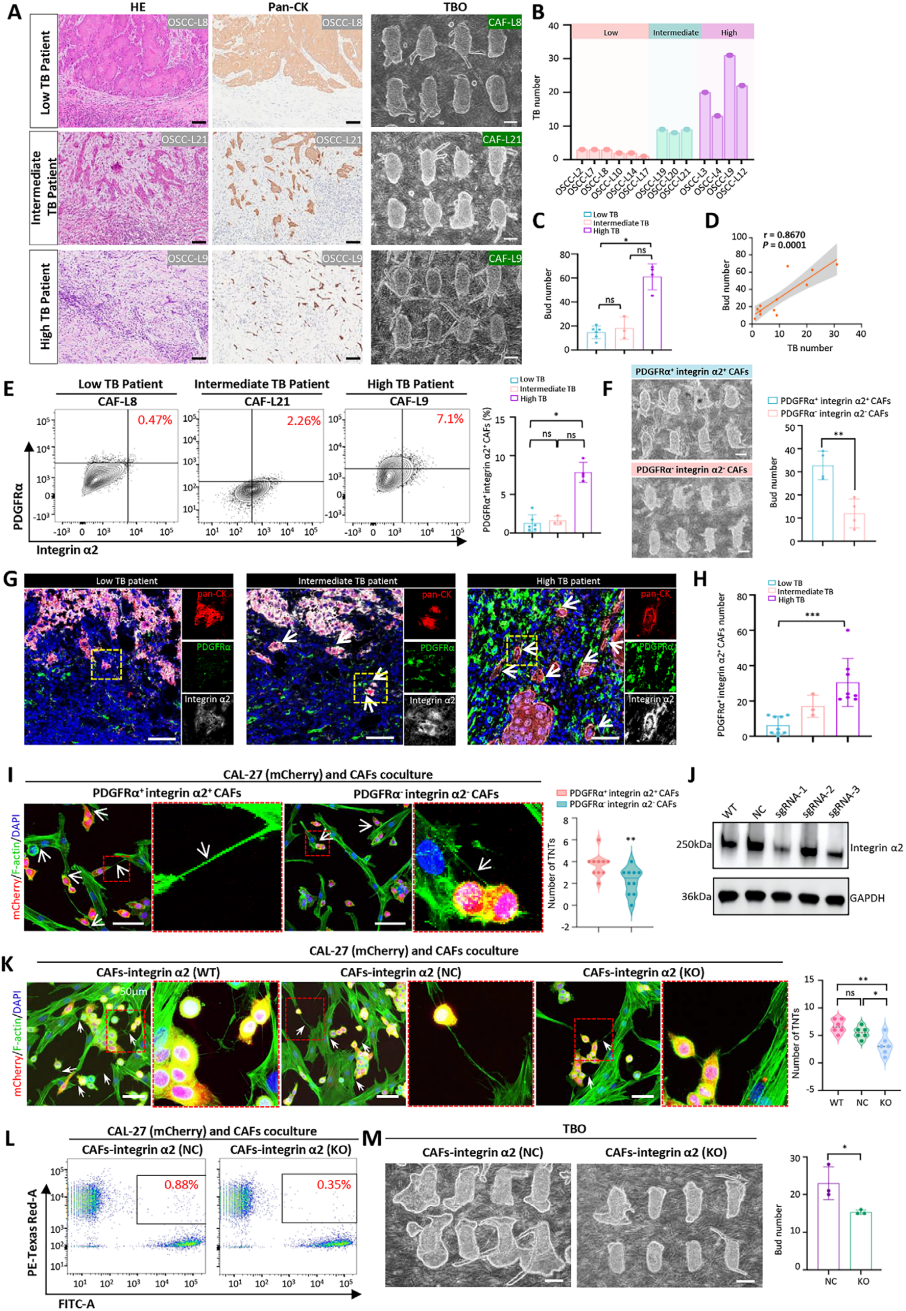
PDGFRα^+^ integrin α2^+^ CAFs are clinically validated as the key subset driving TB formation, with integrin α2‐mediated adhesion promoting TNTs formation and mitochondrial transfer. (A) Representative images of HE and pan‐CK staining in clinical OSCC specimens with high, intermediate, and low TB, alongside corresponding TBO models established using patient‐derived CAFs from each TB group (Scale bars = 100 µm). (B,C) Quantification of tumor buds in pathological sections from patients stratified by TB grade (B) and in the corresponding TBO models (C). Data are presented as mean ± SD. ^*^ *p* < 0.05 by one‐way ANOVA. (D) Correlation analysis of bud number in the TBO model with TB number in patients. (E) Flow cytometric analysis showing the percentage of PDGFRα^+^/integrin α2^+^ CAFs derived from patients with high, intermediate, and low TB. Data are presented as mean ± SD. ^*^ *p* < 0.05 by Kruskal–Wallis test. (F) Representative images of TBO models established by co‐culturing OSCC cells with either PDGFRα^+^/integrin α2^+^ or PDGFRα^−^/integrin α2^−^ CAFs (Scale bars = 100 µm). The right panel shows quantification of tumor buds in the two groups. Data are presented as mean ± SD; *n* = 4 per group. ^**^ *p* < 0.01 by Student's t‐test. (G) Representative images show the expression and co‐localization of integrin α2 (white) and PDGFRα (green) in cancer‐associated fibroblasts (CAFs), while tumor cells are labeled with pan‐CK (red). Nuclei were counterstained with DAPI (blue). Enlarged views of the areas delineated by yellow dashed boxes illustrate PDGFRα^+^ integrin α2^+^ double‐positive CAFs engaged in close contact with adjacent tumor cells (pan‐CK^+^). Scale bars = 50 µm (overview images). (H) Quantification of PDGFRα^+^/integrin α2^+^ CAF numbers in clinical samples stratified by TB grade (high, *n* = 8; intermediate, *n* = 3; low, *n* = 9). Data are presented as mean ± SD. ^***^ *p* < 0.001 by Kruskal‐Wallis test followed by Dunn's post hoc test (high vs. low TB). (I) Immunofluorescence staining showing tunneling nanotubes (TNTs, white arrows) formed between OSCC cells and the indicated CAF subsets after 24 h of co‐culture (Scale bars = 20 µm). Quantification of TNT numbers is shown on the right. Data are mean ± SD (*n* = 3 per group). Statistical significance was determined by student's t‐test (^**^ *p* < 0.01). (J) Western blot validation of integrin α2 knockout efficiency in CAFs using CRISPR‐Cas9. Wild‐type (WT), negative control (NC), and three independent sgRNA‐targeted knockout groups (sgRNA1‐3) are shown. (K) Fluorescence images of CAL‐27 (mCherry) cells co‐cultured for 24 h with CAFs from WT, NC, or integrin α2‐knockout (sgRNA1) groups. White arrows indicate TNT formation between CAFs and CAL‐27 cells (Scale bars = 20 µm). Quantification of TNT numbers per field is presented as mean ± SD; ^*^ *p* < 0.05, ^**^ *p* < 0.01 by one‐way ANOVA. (L) Flow cytometric analysis of CAL‐27 cells after 24 h co‐culture with CAFs transduced with hCOX8A‐EGFP under NC and integrin α2‐knockout (sgRNA1) conditions. The percentage of double‐positive cells (CAL‐27 cells receiving mitochondria from CAFs) is quantified. (M) Bright‐field images of TBO models established by co‐culturing CAL‐27 cells with CAFs from NC and integrin α2‐knockout (sgRNA1) groups (Scale bars = 100 µm). Quantification of tumor buds in each group is shown on the right. Data are presented as mean ± SD; *n* = 3 per group. ^*^ *p* ≤ 0.05 by Student's t‐test.

Multiplex immunofluorescence revealed that PDGFRα^+^/integrin α2^+^ CAFs were frequently located in close proximity to pan‐CK^+^ tumor cells at budding sites (Figure 7G). Quantification further showed that the number of these double‐positive CAFs was significantly higher in high‐TB patients than in low‐TB patients (Figure 7H; Table S2), and correlated positively with TB counts in clinical specimens (Figure S14B). Single‐cell RNA‐seq analysis revealed that the PDGFRA and ITGA2 co‐expression score in the CAF‐C5 subset was significantly higher than that in other CAF subclusters (Figure S14C,D). Moreover, double‐positive CAFs formed substantially more TNTs with CAL‐27 cells after 24 h co‐culture compared to double‐negative controls (Figure 7I).

To validate integrin α2 involvement, we generated integrin α2‐knockout CAFs via CRISPR‐Cas9 (Figure 7J). Knockout CAFs showed markedly reduced TNT formation with CAL‐27 cells compared to WT and NC groups (Figure 7K). Using hCOX8A‐EGFP‐labeled mitochondria, flow cytometry revealed that NC groups exhibited 0.88% double‐positive CAL‐27 cells (indicating mitochondrial transfer), whereas KO group dropped to 0.35% (Figure 7L). Consistently, TBOs established with integrin α2‐deficient CAFs displayed significantly fewer buds than controls (Figure 7M).

Collectively, these findings identify PDGFRα^+^/integrin α2^+^ CAFs as a key pro‐invasive subset in OSCC. Integrin α2‐mediated TNT formation enables mitochondrial transfer from CAFs to cancer cells, and disruption of this interaction impairs both intercellular connectivity and metabolic coupling, highlighting integrin α2 as a potential therapeutic target.

## 3 Discussion

TB is a clinically important prognostic feature in OSCC, yet its stromal regulators and mechanisms remain incompletely defined. Here, we developed a TBO model that recapitulates the cancer‐stroma interface and TB dynamics, enabling identification of PDGFRα^+^/integrin α2^+^ CAFs as key drivers of OSCC budding. Our findings support a mechano‐metabolic symbiosis between these CAFs and OSCC cells that involves: (1) PDGFA/PDGFRα‐mediated recruitment of PDGFRα^+^/integrin α2^+^ CAFs to OSCC nests; (2) heterotypic E‐cadherin/integrin α2β1 adhesion transmitting mechanical cues to activate YAP signaling and induce EMT‐like phenotypes; and (3) TNT‐mediated mitochondrial transfer providing bioenergetic support to budding cells (Figure 8).

**FIGURE 8:**
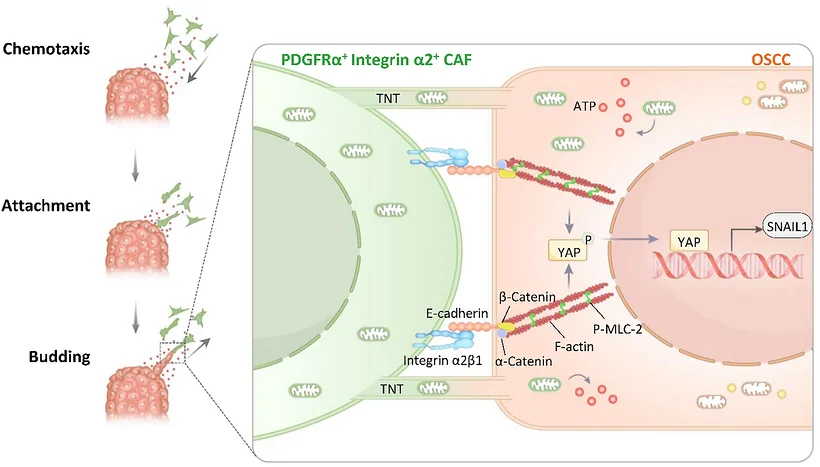
Schematic illustration of CAF‐mediated OSCC budding. Schematic representation of the recruitment of CAFs (green) to OSCC nest (orange) by PDGFA/PDGFRα pathway, followed by CAF‐OSCC contact and subsequent CAF‐mediated mechanical pulling driving OSCC budding invasion. The underlying mechanisms involve CAFs binding to OSCC cells via heterotypic adhesion E‐cadherin/integrin α2β1, and activation of the YAP signaling pathway in OSCC cells to induce EMT. In addition, CAFs transfer mitochondria to OSCC cells via TNTs to supply more ATP for invasion.

The TBO model addresses limitations of traditional in vitro systems by capturing contact‐dependent tumor‐stroma interactions that underlie TB. Tumor spheroids generated in low‐adhesion plates offer limited control over size and morphology, and mechanical stress during transfer can compromise structural integrity [22, 23, 24]. More importantly, spheroids typically lack stromal components and therefore cannot reproduce the CAF‐driven interface required for TB initiation. Conventional OSCC epithelial organoids preserve aspects of tumor cell autonomy, but the absence of CAFs and other TME elements limits their ability to model budding at the invasive front [25]. In contrast, our TBO platform co‐embeds OSCC cells and CAFs within a biomimetic 3D matrix of type I collagen and Matrigel, closely approximating in vivo architecture. This design provides several advantages: (i) real‐time visualization of the full budding sequence, from CAF recruitment and contact to collective or independent budding; (ii) preservation of transcriptional heterogeneity in both CAFs and OSCC cells, as demonstrated by scRNA‐seq; and (iii) clinical relevance, as patient‐derived CAFs reproduce pathological TB grades. Together, these features make TBO a practical platform for mechanistic studies of TB and for screening anti‐budding therapies.

In our TBO model, CAFs drive tumor budding through two distinct modes: collective budding, with CAFs persistently attached at the leading edge, and independent budding, where tumor cells migrate without a CAF companion. Time‐lapse imaging (Video S2) revealed that independent budding often originated from an initial collective phase, during which CAFs detached from budding cells after having led their outward migration. This detachment may result from the higher intrinsic migration speed of CAFs relative to OSCC cells and the possible weakening of heterotypic E‐cadherin/integrin α2β1 adhesion under mechanical stress. The predominance of collective budding suggests that integrin α2β1‐mediated adhesion typically provides sufficient mechanical coupling to maintain CAF‐tumor cell attachment during most budding events. Thus, the relative frequency of the two budding modes likely depends on the balance between CAF motility and adhesive strength.

CAFs are the most abundant and functionally heterogeneous stromal component in the TME, with distinct subtypes exerting divergent effects on tumor progression [26, 27]. Although CAF enrichment in TB regions of OSCC has been reported, the specific CAF subset that drives TB and the underlying mechanisms have remained unclear. Previous studies have attempted to define functional CAF subsets using single markers such as PDGFRα, α‐SMA, or FAP, but these markers lack specificity; PDGFRα^+^ CAFs have been implicated in shaping an immunosuppressive TME [15], whereas α‐SMA^+^ myofibroblastic CAFs are associated with poor prognosis in OSCC [28, 29]. Our study advances this field by identifying a dual‐marker signature (PDGFRα^+^/integrin α2^+^) that specifically defines the TB‐promoting CAF subset. Through scRNA‐seq and functional validation, we showed that CAF‐C5 (characterized by co‐expression of PDGFRα and integrin α2) is the key subtype driving TB and is enriched in pathways related to cell adhesion, ECM remodeling, and pro‐tumorigenic signaling. Importantly, the abundance of PDGFRα^+^/integrin α2^+^ CAFs, but not either marker alone, correlated with TB grade in OSCC patients and induced significantly more budding in TBO models than PDGFRα^−^/integrin α2^−^ CAFs. This dual‐marker signature provides a more precise tool for identifying clinically relevant CAF subsets and may help stratify OSCC patients at high risk for TB‐driven progression.

Prior studies of TB have often focused on individual pathways, including TGF‐β‐mediated EMT [30], MMP‐dependent ECM degradation [31], and hypoxia‐induced metabolic reprogramming [32]. While these pathways contribute to invasion, they do not fully capture the coordinated crosstalk between cancer cells and stromal components that enables TB. Our findings support a mechanometabolic symbiosis between PDGFRα^+^/integrin α2^+^ CAFs and OSCC cells that integrates distinct mechanical and metabolic inputs to promote budding.

Cell‐cell adhesion molecules play a central role in mediating cancer‐stroma crosstalk and invasion. Heterotypic E‐cadherin/N‐cadherin adhesion between cancer cells and CAFs has been reported to promote invasion in 2D cultures [33], but whether heterotypic adhesion complexes function in 3D environments to drive TB has remained unclear. Using ligand‐receptor analysis and functional validation, we demonstrated that PDGFRα^+^/integrin α2^+^ CAFs interact with OSCC cells through heterotypic E‐cadherin/integrin α2β1 adhesion. This interaction is associated with cytoskeletal remodeling (α‐catenin/β‐catenin colocalization and elevated p‐MLC‐2 expression) and activation of YAP signaling, a key mediator of mechanical stress responses. YAP activation coincides with a partial EMT‐like phenotype in budding OSCC cells, including nuclear localization of SNAIL‐1, enhanced migratory capacity, and retention of E‐cadherin expression. Such hybrid epithelial‐mesenchymal states may balance motility with survival during budding.

Mitochondrial transfer has emerged as a mechanism of metabolic crosstalk in the TME, allowing cancer cells to overcome metabolic stress and acquire bioenergetic support for invasion and metastasis [34]. In this study, OSCC cells displayed structurally and functionally impaired mitochondria with reduced ATP production capacity, whereas PDGFRα^+^/integrin α2^+^ CAFs contained intact mitochondria and higher respiratory capacity. We demonstrated that these CAFs transfer functional mitochondria to OSCC cells via TNTs, enhancing OXPHOS and ATP production in recipient cells, particularly within buds. Notably, PDGFRα^+^/integrin α2^+^ CAFs formed more TNTs with OSCC cells than PDGFRα^−^/integrin α2^−^ CAFs, suggesting that E‐cadherin/integrin α2β1 adhesion may facilitate TNT formation. Together, these findings highlight a coordinated mechanical‐adhesion and metabolic‐transfer program underlying the mechano‐metabolic symbiosis driving TB.

Our findings suggest several actionable nodes for inhibiting TB, including PDGFRα signaling, integrin α2β1‐mediated adhesion, and TNT‐mediated mitochondrial transfer. Imatinib, an FDA‐approved PDGFRα inhibitor, suppressed CAF‐induced TB in the TBO model, suggesting opportunities for drug repurposing in TB‐high OSCC. Inhibitors targeting integrin α2β1 are under development, and our data provide a rationale for evaluating these agents in anti‐budding strategies. In addition, TNT inhibitors such as L‐778123 represent a tractable approach to disrupt metabolic support from CAFs. Beyond therapy, the PDGFRα^+^/integrin α2^+^ CAF signature may serve as a prognostic biomarker to identify patients at elevated risk for TB‐driven progression and to stratify patients for targeted interventions.

This study has limitations. First, the current TBO model lacks immune components of the TME, which may regulate CAF function and TB; incorporating immune cells (e.g., macrophages and T cells) would provide a more comprehensive microenvironmental context. Second, while we focused on PDGFRα^+^/integrin α2^+^ CAFs, other CAF subsets may contribute to TB in a context‐dependent manner, and future studies should explore interactions among CAF subtypes. Third, our clinical validation was based on a relatively small cohort of 10 OSCC patients; expanding to larger cohorts with long‐term follow‐up will strengthen clinical interpretation. Finally, the molecular coupling between E‐cadherin/integrin α2β1 adhesion and TNT formation remains to be elucidated, and future work should define the downstream signaling that links these processes.

In conclusion, we establish a tumor‐budding organoid model that captures CAF‐driven OSCC budding and identify PDGFRα^+^/integrin α2^+^ CAFs as central regulators of this phenotype through coordinated mechanical and metabolic crosstalk. The dual‐marker signature and the two interconnected crosstalk pathways (heterotypic E‐cadherin/integrin α2β1 adhesion and TNT‐mediated mitochondrial transfer) suggest actionable targets for developing anti‐budding therapies, while the TBO platform provides a tractable system for mechanistic studies and drug screening. Together, these findings advance understanding of TB regulation and may inform improved clinical management of OSCC patients with high TB grade.

## 4 Materials and Methods

### 4.1 Establishment of TBOs

The mold for polydimethylsiloxane (PDMS) stamp was fabricated by photolithography using SU8 2050 (Microchem). PDMS prepolymer and curing reagent (Sylgard 184 Silicone Elastomer Kit) were mixed in a 10:1 ratio, poured onto the mold, and cured in an oven at 80°C for 1 h. After cooling, the PDMS was gently peeled off the mold and trimmed to size. PDMS was cut into 5 × 5 cm pieces (referred to as PDMS stamps), each containing 36 microwells capable of holding cells. The PDMS stamp was coated with BSA (1% w/v in PBS; Sigma–Aldrich) overnight to prevent collagen adhesion. The BSA solution was aspirated from the stamp, and the BSA‐coated surface was washed twice with a mixture of collagen I (3 mg/mL, R&D, 3440‐100‐01) and Matrigel (Corning, 354234) at a volume ratio of 3:1. A mixture of collagen I and Matrigel with or without CAFs at a density of 2.5–3 × 10^5^ cells/mL was added to the surface of the PDMS stamp. The stamp was then flipped over into a 35‐mm dish. The dish was put in a 37 C incubator for 1 h to allow the gel gelation. The stamp was carefully removed from the gel, and microwells were formed on the gel surface. Tumor cells (10^6^–10^7^ cells/mL) were then added to the surface of the gel and allowed to settle within the microwells for approximately 2 min. Non‐adherent cells were gently washed with medium, and the dish was placed in at 37 C incubator for 10 min. Then, a collagen slide was prepared by pipetting 30 µL of collagen I‐containing CAFs onto the surface of a sterile coverslip and covering the gel surface. The cell culture medium was then added.

### 4.2 Single‐Cell Dissociation of Organoids

Organoids were treated with TrypLE (ThermoFisher) in combination with 1 mg/mL Collagenase IV (Worthington) and 1 mm CaCl_2_ (Sigma) at 37°C for 30 min, with pipetting every 5 min. The cell clusters were filtered through a 40‐micron cell strainer (BeyoGold, FSTR040), centrifuged, and resuspended in PBS.

### 4.3 Single‐Cell RNA Sequencing (ScRNA‐Seq)

The Chromium Single‐Cell 5′ Library and Gel Bead Kit (10× Genomics) was used in accordance with the manufacturer's protocols. Briefly, gel bead‐based emulsions were generated by combining barcoded single‐cell 5′ Gel Beads, cells, and partitioning oils. Cells were lysed within individual oil droplets, where the mRNA poly(A) tails were tagged with cell‐specific barcodes on the surface of the beads, and reverse transcription was subsequently performed to generate cDNA. Enriched libraries were enzymatically digested, size‐selected, and adaptor‐ligated for sequencing. Sequencing libraries were generated using unique sample indices for each sample and quantified using the Kapa library kit. The quantified libraries were sequenced using the Illumina NovaSeq 6000 Sequencing System (RRID: SCR_016387).

### 4.4 In Situ RNA Sequencing (isRNA‐Seq)

Organoids were embedded in Optimal Cutting Temperature compound (SAKURA, 4583) and sectioned into 10 µm slices using a cryostat (Leica CM1860, RRID: SCR_025772). Before RNA‐seq, sections were fixed in 4% paraformaldehyde in diethyl pyrocarbonate (DEPC)‐treated PBS (DEPC‐PBS) for 5 min at room temperature and then washed twice in DEPC‐PBS. the sections were permeabilized with 0.1 m HCl at 37°C for 5 min and incubated in the following order: target probes with a specific barcode for each RNA molecule in hybridization mix at 37°C for 4 h, ligation mix at 37°C for 30 min, splint primers in the circularization mix at 37°C for 30 min, and amplification mix for rolling circle amplification (RCA) at 30°C overnight. After each step, the slides were washed three times with Wash Buffer at room temperature. For sequencing, the sections were incubated in the ligation buffer of anchor primers, label sequencing interrogation probes, and ligase at 30°C for 45 min, followed by mounting with SlowFade Gold Antifade Mountant (Thermo Fisher Scientific, Shanghai, China) medium containing 0.5 µg/mL DAPI (Sigma–Aldrich, D9542).

### 4.5 Flow Cytometry

Cells were suspended in BD Pharmingen Stain Buffer (Catalog No.554657) and centrifuged at 300 g for 5 min at 4°C. The final cell concentration was adjusted to 2 × 10^7^ cells/mL using pre‐cooled BD Pharmingen Stain Buffer. Transfer 100 µL of the cell suspension (containing 10^6^ cells) to a 1.5 mL round‐bottom polypropylene tube. APC‐PDGFRα (5 µL/test; BD 562798, RRID: AB_2737803), FITC‐integrin α2 (20 µL/test; BD555498, RRID: AB_395888), or Alexa Fluor 488‐Integrin β1(1/500; ab193591, RRID: AB_3720984) were added to each tube. The mixture was then incubated on ice in the dark for 20 min. The cells were washed twice with an appropriate volume of Stain Buffer (1 mL per tube) and centrifuged at 300 × g for 5 min. The supernatant was carefully removed. The cells were resuspended in an appropriate volume of Stain Buffer and analyzed using flow cytometry (BD FACSAria III, RRID: SCR_016695). Live single cells labeled with the PDGFRα antibody were sorted using flow cytometry.

### 4.6 PDGFRα Blocking

After TBO construction, imatinib (MCE, HY‐15463), a selective tyrosine kinase inhibitor targeting BCR/ABL, v‐Abl, PDGFR, and c‐kit kinases, was added to the culture medium by binding to their ATP‐binding sites. The cells were maintained under standard culture conditions for 5 days. The budding invasion of OSCC cells was observed under a microscope.

### 4.7 Mitochondrial Transfer Assay

For coculture experiments, hCOX8A‐EGFP‐transduced CAFs were cocultured with untransduced OSCC cells, or conversely, hCOX8A‐EGFP‐transduced OSCC cells were cocultured with untransduced CAFs. After 24 h of coculture, cells were fixed with 4% paraformaldehyde for 20 min at room temperature, followed by three washes with PBS (15 min each). To visualize tunneling nanotubes (TNTs), cells were stained with Actin‐Tracker Red‐Rhodamine (1:400; Beyotime, C2207S) or Actin‐Tracker Green‐488 (1:400; Beyotime, C2201S) for 1 h at room temperature. Nuclei were counterstained with DAPI (D9542; Sigma–Aldrich) for 15 min. Fluorescence images were acquired using a Leica DMi8 confocal microscope (Leica Microsystems, RRID: SCR_024663).

### 4.8 Mitochondrial Transfer Blocking

TNTs formation was inhibited by pretreatment with L‐778123 (MCE, HY‐16273A), a dual inhibitor of farnesyltransferase and geranylgeranyltransferase 1, or cytochalasin B (SPARKJADE, SJ‐MN0899), a cytoskeleton‐disrupting agent. Both inhibitors were added at the start of TBO model establishment (0 h). Cultures were maintained under standard conditions for five days, and mitochondrial transfer inhibition was assessed via fluorescence microscopy on day five.

### 4.9 ATP Staining

In 2D culture systems, intracellular ATP levels in individual cell types (CAFs or OSCC cells) were measured as follows. Cells were suspended and seeded into 24‐well plates. Upon reaching 60%–80% confluence, the culture medium was removed and the cells were washed three times with PBS. BioTracker ATP Live Cell Dye (Sigma, SCT045) was added, followed by incubation for 30 min under standard culture conditions. ATP signals were visualized by fluorescence microscopy. To investigate whether ATP was transferred from CAFs to OSCC cells during mitochondrial trafficking, hCOX8A‐EGFP‐labeled CAFs were co‐cultured with CAL‐27 cells for 24 h. Following co‐culture, ATP was detected using the protocol described above. For the TBO model, TBO was constructed for 5 days, the medium was removed, the cells were rinsed three times with PBS (5 min each), ATP reagent was added, the cells were incubated for 30 min, and ATP levels were observed under a confocal fluorescence microscope.

### 4.10 Seahorse Metabolic Assay

hCOX8A‐EGFP‐labeled CAFs and CAL‐27 cells were co‐cultured at a 1:1 ratio for 48 h, followed by digestion into a single‐cell suspension and staining with E‐cadherin (APC‐conjugated, Proteintech, APC‐FcA98123, RRID: AB_3720985) for 20 min on ice; APC^+^ and FITC^+^APC^+^ cells were then sorted by FACS and cultured for 5 days prior to the Seahorse metabolic assay. One day before the assay, sorted cells and parental CAL‐27 cells without any CAF exposure were seeded into a Seahorse XFe96 plate (103794‐100, Agilent) at a density of 2 × 10^4^ cells per well, while the Seahorse XFe96/XF Pro FluxPak Mini (103793‐100, Agilent) and calibration solution (100840‐000, Agilent) were equilibrated overnight in a CO2‐free incubator. On the day of the assay, the medium was replaced with XF DMEM Base Medium (103575‐100, Agilent) supplemented with 10 mm glucose (103577‐100, Agilent), 1 mm pyruvate (103578‐100, Agilent), and 2 mm glutamine (103579‐100, Agilent). Mitochondrial function was assessed using the Seahorse XF Cell Mito Stress Test Kit (103015‐100‐6ea, Agilent) with sequential injections of 1.5 µm oligomycin, 2.0 µm FCCP, and 0.5 µm rotenone/antimycin A following the manufacturer's instructions.

### 4.11 Grading of TB Assessment

The evaluation of TB grade mainly adopted HE‐stained sections. Immunohistochemical staining against pan‐CK was used for auxiliary evaluation. The assessment was performed at the invasive front by selecting a single hotspot with the highest bud density under a 20× objective lens (field area: 0.785 mm^2^). The TB grade was assigned according to the bud count in the highest‐density hotspot as follows: low‐grade (0‐4 buds), intermediate‐grade (5‐9 buds), and high‐grade (≥10 buds). Histopathological evaluation and scoring of H&E and IHC‐stained tissue sections were performed by two independent pathologists blinded to all clinical and experimental group information.

### 4.12 Image Analysis and Quantification of PDGFRα^+^ CAFs

Multicolor immunofluorescent images of OSCC tissue sections were analysed using the HALO image analysis platform (Indica Labs, USA, RRID: SCR_018350). Briefly, the region of interest (ROI) was first manually delineated around the tumor tissue. Subsequently, the High‐Plex Analysis Module within HALO was employed for cell quantification. A specific fluorescence signature for the PDGFRα marker was established by manually selecting positive signals across the appropriate channel. This color threshold was rigorously optimized and consistently applied to all samples stained for the same marker to ensure reproducible quantification. The software algorithm identified all cell nuclei based on DAPI signal and performed cytoplasmic expansion to define individual cell boundaries. PDGFRα^+^ CAFs were classified as cells negative for epithelial and immune lineage markers but positive for PDGFRα within the stromal compartments. The abundance of PDGFRα^+^ CAFs was quantified and expressed as the number of positive cells per square millimetre (number/mm^2^).

### 4.13 Time‐Lapse Imaging

After 24 h of co‐culture with CAFs and OSCC cells, their interactions were observed using a fully automated live‐cell imaging system (CELLImage Mini, CIMB1000) for a total duration of 72 h, with imaging intervals of 2 h. After monitoring, the cell images were examined, and integrated image analysis was performed.

### 4.14 Animal Models

To establish orthotopic xenograft models of OSCC, 6‐week‐old male BALB/c nude mice (RRID: MGI:2161072) were used. CAL‐27 cells (8 × 10^5^), with or without CAFs (4 × 10^5^), were injected into the anterior portion of the tongue using a 30‐gauge needle syringe (Kindly U‐100). Seven experimental groups were designed: (1) CAL‐27 alone; (2–5) CAL‐27 + CAF‐L1/L2/L3/L4; (6‐7) CAL‐27 + CAF‐L2/L4 + L‐778123 (Selleck, E4620). L‐778123 was administered every 48 h starting at 72 h post‐implantation (120 mg/kg). Mouse weight was monitored daily. Tumors were measured every alternate day using a Vernier caliper. The tumor volume (Vt) was calculated using the following formula: L × B^2^/2, where L is the longest dimension, and B is the shortest dimension. All animal treatment procedures were approved by the Animal Care and Use Committee of Shanghai Jiao Tong University and were performed in accordance with the institutional guidelines.

### 4.15 Statistical Analyses

Statistically significant differences between the two groups were assessed using the Student's t‐test. For comparisons between two groups, if the data followed a normal distribution, an unpaired Student's t‐test was applied; otherwise, the non‐parametric Mann–Whitney test was used. For comparisons involving more than two experimental groups, one‐way ANOVA followed by Bonferroni's multiple comparison test was performed when data met the assumptions of normality and homogeneity of variance. In cases where sample sizes were small, or data did not follow a normal distribution, particularly for comparisons among three or more unpaired groups, the non‐parametric Kruskal–Wallis test was applied, followed by Dunn's multiple comparisons test where appropriate. Pearson's correlation coefficient was used to assess the correlation between paired variables. The correlation strength was evaluated based on the correlation coefficient (r) and the corresponding *p*‐value. All statistical analyses were performed using GraphPad Prism software (version 10.0, RRID: SCR_002798). All experiments were repeated at least three times to ensure reproducibility and statistical fidelity. Statistical significance was set at *p* < 0.05.

## Author Contributions

T.L. and T.J. supervised the project, conceived of the study, designed the experiments, and wrote the manuscript. Y.L. and J.L. analyzed the single‐cell sequencing data, performed in vitro and in vivo experiments, and contributed to manuscript writing. J.L. analyzed clinical data. H.Z. performed the immunohistochemistry and hematoxylin‐eosin staining. M.L., H.L., and P.L. recruited patients and provided clinical samples. Z.Y. performed the TCGA data analysis. Q.D., Y.G., L.C., and M.Z. performed animal experiments. F.G., H.L., X.L., and Y.W. participated in data analysis. All authors interpreted the results and critically reviewed and edited the manuscript. All the authors have read and approved the final version of the manuscript.

## Ethics Statements

Human tumor samples were collected from treatment‐naive patients with OSCC at Shanghai Stomatological Hospital (Ethics Approval No. [2024]023), and written informed consent was obtained from all the participants. Animal experiments, including housing, transportation, and care, were conducted in accordance with relevant regulations and guidelines approved by the Animal Ethics Committee of Shanghai Jiao Tong University (Approval No. A2025010). All original, uncropped immunoblot images are provided in the Supplementary Information.

## Conflicts of Interest

The authors declare no conflicts of interest.

## Supporting information




**Supporting File 1**: advs76385‐sup‐0001‐SuppMat.docx.

**Supporting File 2**: advs76385‐sup‐0002‐DataFile.docx.

**Supporting File 3**: advs76385‐sup‐0003‐TableS1.docx.

**Supporting File 4**: advs76385‐sup‐0004‐TableS2.docx.

**Supporting File 5**: advs76385‐sup‐0005‐VideoS1.mp4.

**Supporting File 6**: advs76385‐sup‐0006‐VideoS2.mp4.

## Data Availability

The single‐cell transcriptomic sequencing data from this study have been submitted and are housed in the NCBI Sequence Read Archive (SRA) database under the permanent BioProject accession PRJNA1403221 (https://www.ncbi.nlm.nih.gov/sra/PRJNA1403221). Individual sample metadata can be accessed through the linked BioSample accessions SAMN54622777 and SAMN54622778. Additional datasets are available from the corresponding author upon reasonable request.

## References

[1] A. C. Chi , T. A. Day , and B. W. Neville , “Oral Cavity and Oropharyngeal Squamous Cell Carcinoma–an Update,” CA: A Cancer Journal for Clinicians 65 (2015): 401–421. 10.3322/caac.21293 26215712

[2] Y.‐F. Yu , L.‐M. Cao , Z.‐Z. Li , et al., “Frequency of Lymph Node Metastases at Different Neck Levels in Patients With Oral Squamous Cell Carcinoma: a Systematic Review and Meta‐analysis,” International Journal of Surgery 111 (2025): 1285–1300, 10.1097/JS9.0000000000001953. PMC11745673 39037727

[3] A. Lugli , I. Zlobec , M. D. Berger , R. Kirsch , and I. D. Nagtegaal , “Tumour Budding in Solid Cancers,” Nature Reviews Clinical Oncology 18 (2021): 101–115, 10.1038/s41571-020-0422-y. 32901132

[4] C. G. Attramadal , S. Kumar , M. E. Boysen , H. P. Dhakal , J. M. Nesland , and M. Bryne , “Tumor Budding, EMT and Cancer Stem Cells in T1‐2/N0 Oral Squamous Cell Carcinomas,” Anticancer Research 35 (2015): 6111–6120. 26504037

[5] M. Gonzalez‐Guerrero , P. Martínez‐Camblor , B. Vivanco , et al., “The Adverse Prognostic Effect of Tumor Budding on the Evolution of Cutaneous Head and Neck Squamous Cell Carcinoma,” Journal of the American Academy of Dermatology 76 (2017): 1139–1145, 10.1016/j.jaad.2017.01.015. 28314684

[6] A. A. Makitie , A. Almangush , J. P. Rodrigo , A. Ferlito , and I. Leivo , “Hallmarks of Cancer: Tumor Budding as a Sign of Invasion and Metastasis in Head and Neck Cancer,” Head & Neck 41 (2019): 3712–3718, 10.1002/hed.25872. 31328847

[7] A. Lugli , R. Kirsch , Y. Ajioka , et al., “Recommendations for Reporting Tumor Budding in Colorectal Cancer Based on the International Tumor Budding Consensus Conference (ITBCC) 2016,” Modern Pathology 30 (2017): 1299–1311, 10.1038/modpathol.2017.46. 28548122

[8] L. Togni , V. C. A. Caponio , N. Zerman , et al., “The Emerging Impact of Tumor Budding in Oral Squamous Cell Carcinoma: Main Issues and Clinical Relevance of a New Prognostic Marker,” Cancers (Basel) 14 (2022): 3571. 10.3390/cancers14153571 PMC9332070 35892830

[9] Y. Zhu , H. Liu , N. Xie , et al., “Impact of tumor budding in head and neck squamous cell carcinoma: A meta‐analysis,” Head & Neck 41 (2019): 542–550, 10.1002/hed.25462. 30549142

[10] A. Almangush , M. Pirinen , I. Heikkinen , A. A. Makitie , T. Salo , and I. Leivo , “Tumour Budding in Oral Squamous Cell Carcinoma: a Meta‐analysis,” British Journal of Cancer 118 (2018): 577–586, 10.1038/bjc.2017.425. PMC5830589 29190636

[11] M. Raudenska , J. Balvan , K. Hanelova , M. Bugajova , and M. Masarik , “Cancer‐associated Fibroblasts: Mediators of Head and Neck Tumor Microenvironment Remodeling,” Biochimica et Biophysica Acta (BBA)—Reviews on Cancer 1878 (2023): 188940, 10.1016/j.bbcan.2023.188940. 37331641

[12] A. T. Ruffin , H. Li , L. Vujanovic , D. P. Zandberg , R. L. Ferris , and T. C. Bruno , “Improving Head and Neck Cancer Therapies by Immunomodulation of the Tumour Microenvironment,” Nature Reviews Cancer 23 (2023): 173–188, 10.1038/s41568-022-00531-9. PMC9992112 36456755

[13] E. Sahai , I. Astsaturov , E. Cukierman , et al., “A Framework for Advancing Our Understanding of Cancer‐associated Fibroblasts,” Nature Reviews Cancer 20 (2020): 174–186, 10.1038/s41568-019-0238-1. PMC7046529 31980749

[14] S. Rajthala , A. Min , H. Parajuli , et al., “Profiling and Functional Analysis of microRNA Deregulation in Cancer‐Associated Fibroblasts in Oral Squamous Cell Carcinoma Depicts an Anti‐Invasive Role of microRNA‐204 via Regulation of Their Motility,” International Journal of Molecular Sciences 22 (2021): 11960, 10.3390/ijms222111960. PMC8584862 34769388

[15] H. Jia , X. Chen , L. Zhang , and M. Chen , “Cancer Associated Fibroblasts in Cancer Development and Therapy,” Journal of Hematology & Oncology 18 (2025): 36, 10.1186/s13045-025-01688-0. PMC11954198 40156055

[16] D. S. Foster , M. Januszyk , D. Delitto , et al., “Multiomic Analysis Reveals Conservation of Cancer‐associated Fibroblast Phenotypes Across Species and Tissue of Origin,” Cancer Cell 40 (2022): 1392–1406.e7, 10.1016/j.ccell.2022.09.015. PMC9669239 36270275

[17] Y. Chen , K. M. McAndrews , and R. Kalluri , “Clinical and Therapeutic Relevance of Cancer‐associated Fibroblasts,” Nature Reviews Clinical Oncology 18 (2021): 792–804, 10.1038/s41571-021-00546-5. PMC8791784 34489603

[18] Y. Liu , A. Sinjab , J. Min , et al., “Conserved Spatial Subtypes and Cellular Neighborhoods of Cancer‐associated Fibroblasts Revealed by Single‐cell Spatial Multi‐omics,” Cancer Cell 43 (2025): 905–924.e6, 10.1016/j.ccell.2025.03.004. PMC12074878 40154487

[19] P. Zhuang , Y. H. Chiang , M. S. Fernanda , and M. He , “Using Spheroids as Building Blocks towards 3D Bioprinting of Tumor Microenvironment,” International Journal of Bioprinting 7 (2021): 444, 10.18063/ijb.v7i4.444. PMC8600307 34805601

[20] B. Chen , Y. Wu , Z. Ao , et al., “High‐throughput Acoustofluidic Fabrication of Tumor Spheroids,” Lab on a Chip 19 (2019): 1755–1763, 10.1039/C9LC00135B. 30918934

[21] S. H. Kang , S. Y. Oh , H. J. Lee , et al., “Cancer‐Associated Fibroblast Subgroups Showing Differential Promoting Effect on HNSCC Progression,” Cancers 13 (2021): 654. 10.3390/cancers13040654 PMC7915931 33562096

[22] S. Gan , D. G. Macalinao , S. H. Shahoei , et al., “Distinct Tumor Architectures and Microenvironments for the Initiation of Breast Cancer Metastasis in the Brain,” Cancer Cell 42 (2024): 1693–1712.e24, 10.1016/j.ccell.2024.08.015. PMC12093277 39270646

[23] K. Sato , W. Zhang , S. Safarikia , et al., “Organoids and Spheroids as Models for Studying Cholestatic Liver Injury and Cholangiocarcinoma,” Hepatology 74 (2021): 491–502, 10.1002/hep.31653. PMC8529583 33222247

[24] K. Bialkowska , P. Komorowski , M. Bryszewska , and K. Milowska , “Spheroids as a Type of Three‐Dimensional Cell Cultures—Examples of Methods of Preparation and the Most Important Application,” International Journal of Molecular Sciences 21 (2020): 6225, 10.3390/ijms21176225. PMC7503223 32872135

[25] E. Driehuis , S. Kolders , S. Spelier , et al., “Oral Mucosal Organoids as a Potential Platform for Personalized Cancer Therapy,” Cancer Discovery 9 (2019): 852–871, 10.1158/2159-8290.CD-20-0129. 31053628

[26] L. Cords , N. de Souza , and B. Bodenmiller , “Classifying Cancer‐associated Fibroblasts—The Good, the Bad, and the Target,” Cancer Cell 42 (2024): 1480–1485, 10.1016/j.ccell.2024.08.011. 39255773

[27] C. Hu , Y. Zhang , C. Wu , and Q. Huang , “Heterogeneity of Cancer‐associated Fibroblasts in Head and Neck Squamous Cell Carcinoma: Opportunities and Challenges,” Cell Death Discovery 9 (2023): 124, 10.1038/s41420-023-01428-8. PMC10102018 37055382

[28] L. Ding , Z. Zhang , D. Shang , et al., “α‐Smooth Muscle Actin‐Positive Myofibroblasts, in Association With Epithelial–Mesenchymal Transition and Lymphogenesis, is a Critical Prognostic Parameter in Patients With Oral Tongue Squamous Cell Carcinoma,” Journal of Oral Pathology & Medicine 43 (2014): 335–343, 10.1111/jop.12143. 24313357

[29] M. V. Shete , A. V. Shete , K. Buva , P. P. Channe , R. Sapkal , and A. N. Rajbhoj , “Prognostic Ability of Expression of Myofibroblasts in Oral Squamous Cell Carcinoma: a Systematic Review and Meta‐Analysis,” Asian Pacific Journal of Cancer Prevention 25 (2024): 1477–1486, 10.31557/APJCP.2024.25.5.1477. PMC11318827 38809619

[30] D. Jensen , E. Dabelsteen , L. Specht , et al., “Molecular Profiling of Tumour Budding Implicates TGFβ‐mediated Epithelial–mesenchymal Transition as a Therapeutic Target in Oral Squamous Cell Carcinoma,” The Journal of Pathology 236 (2015): 505–516, 10.1002/path.4550. 25925492

[31] C. A. Rubio , “Further Studies on the Histological Characteristics Linked to Local Tumour Invasion in Colorectal Carcinomas,” Anticancer Research 23 (2003): 3555–3560. 12926106

[32] A. Righi , I. Sarotto , L. Casorzo , S. Cavalchini , E. Frangipane , and M. Risio , “Tumour Budding Is Associated With Hypoxia at the Advancing front of Colorectal Cancer,” Histopathology 66 (2015): 982–990, 10.1111/his.12602. 25381897

[33] A. Labernadie , T. Kato , A. Brugués , et al., “A Mechanically Active Heterotypic E‐cadherin/N‐cadherin Adhesion Enables Fibroblasts to Drive Cancer Cell Invasion,” Nature Cell Biology 19 (2017): 224–237, 10.1038/ncb3478. PMC5831988 28218910

[34] V. Marabitti , E. Vulpis , F. Nazio , and S. Campello , “Mitochondrial Transfer as a Strategy for Enhancing Cancer Cell Fitness: Current Insights and Future Directions,” Pharmacological Research 208 (2024): 107382, 10.1016/j.phrs.2024.107382. 39218420

