# Supplementary material for: PDGFRα+/Integrin α2+ Fibroblasts Orchestrate Tumor Budding in Oral Squamous Cell Carcinoma via Mechano‐Metabolic Symbiosis: E‐Cadherin/Integrin α2β1 Adhesion and Mitochondrial Transfer
Source: Adv Sci (Weinh). 2026 Jun 30;13(54):e76385. doi: 10.1002/advs.76385 (PMC13337076; doi:10.1002/advs.76385)
Supplement: Supplementary file 2 [file ADVS-13-e76385-s003.docx]

**Original uncropped Western blot images presented in this study.**

**36 kDa**

**Integrin α2**

**WT**

**GAPDH**

**NC**

**sgRNA-1**

**36 kDa**

**250 kDa**

**sgRNA-2**

**sgRNA-3**

**150 kDa**

**14 kDa**

**CAFs-2**

**CAL-27**

**UM-SCC-1**

**CAFs-1**

**150kDa**

**14kDa**

**PDGFRα**

**PDGFA**

**GAPDH**

**36kDa**
