# Supplementary material for: PDGFRα+/Integrin α2+ Fibroblasts Orchestrate Tumor Budding in Oral Squamous Cell Carcinoma via Mechano‐Metabolic Symbiosis: E‐Cadherin/Integrin α2β1 Adhesion and Mitochondrial Transfer
Source: Adv Sci (Weinh). 2026 Jun 30;13(54):e76385. doi: 10.1002/advs.76385 (PMC13337076; doi:10.1002/advs.76385)
Supplement: Supplementary file 3 [file ADVS-13-e76385-s004.docx]

**Table S1.** **Clinical information of OSCC tissues used for primary CAF isolation.**

| **Tissues** | **Patients ID** | **Gender** | **Age** | **Site** | **CAF** |
| --- | --- | --- | --- | --- | --- |
| OSCC | 1 | Male | 73 | Floor of mouth and tongue | CAF-L2 |
| OSCC | 2 | Female | 81 | Right tongue | CAF-L3 |
| OSCC | 3 | Male | 52 | Left tongue | CAF-L4 |
| OSCC | 4 | Male | 67 | Right Buccal Mucosa | CAF-L7 |
| OSCC | 5 | Male | 38 | Left Buccal Mucosa | CAF-L8 |
| OSCC | 6 | Male | 45 | Left tongue | CAF-L9 |
| OSCC | 7 | Male | 67 | Right tongue | CAF-L10 |
| OSCC | 8 | Male | 50 | Right tongue | CAF-L12 |
| OSCC | 9 | Female | 67 | Left maxillary gingiva | CAF-L14 |
| OSCC | 10 | Male | 69 | Right upper gingiva | CAF-L17 |
| OSCC | 11 | Male | 24 | Right tongue | CAF-L19 |
| OSCC | 12 | Male | 41 | Right upper gingiva | CAF-L20 |
| OSCC | 13 | Female | 73 | Right Buccal Mucosa | CAF-L21 |

Abbreviations: OSCC, Oral squamous cell carcinoma
