# Supplementary material for: PDGFRα+/Integrin α2+ Fibroblasts Orchestrate Tumor Budding in Oral Squamous Cell Carcinoma via Mechano‐Metabolic Symbiosis: E‐Cadherin/Integrin α2β1 Adhesion and Mitochondrial Transfer
Source: Adv Sci (Weinh). 2026 Jun 30;13(54):e76385. doi: 10.1002/advs.76385 (PMC13337076; doi:10.1002/advs.76385)
Supplement: Supplementary file 4 [file ADVS-13-e76385-s005.docx]

**Table S2.** **Clinical information of OSCC tissues used for multiplex immunofluorescence staining.**

| **Patients ID** | **Gender** | **Age** | **Site** | **TB grade** | **TB number** | **PDGFRα⁺/integrin α2⁺ CAF number** |
| --- | --- | --- | --- | --- | --- | --- |
| 1 | Male | 76 | lower lip | Low | 1 | 11 |
| 2 | Male | 66 | Right Buccal Mucosa | High | 12 | 21 |
| 3 | Male | 37 | Left Buccal Mucosa | Low | 4 | 12 |
| 4 | Male | 23 | upper lip and gingiva | Low | 0 | 4 |
| 5 | Male | 69 | Right upper gingiva | Low | 1 | 12 |
| 6 | Male | 70 | Right lower gingiva | Low | 2 | 2 |
| 7 | Female | 82 | Left tongue | Low | 0 | 0 |
| 8 | Male | 24 | Right tongue | High | 11 | 38 |
| 9 | Male | 41 | Right upper gingiva | Intermediate | 7 | 24 |
| 10 | Female | 73 | Right Buccal Mucosa | High | 20 | 24 |
| 11 | Male | 51 | Floor of the mouth mucosa | Intermediate | 5 | 12 |
| 12 | Male | 43 | Right lower gingiva | Intermediate | 6 | 15 |
| 13 | Male | 50 | Right tongue | High | 12 | 35 |
| 14 | Male | 45 | Right tongue | High | 30 | 60 |
| 15 | Male | 40 | Left tongue | High | 10 | 23 |
| 16 | Male | 63 | Right tongue | High | 28 | 22 |
| 17 | Male | 80 | Right lower gingiva | High | 29 | 21 |
| 18 | Female | 67 | Left maxillary gingiva | Low | 4 | 11 |
| 19 | Male | 73 | Floor of the mouth mucosa | Low | 1 | 2 |
| 20 | Male | 67 | Right tongue | Low | 4 | 2 |
