# Supplementary material for: PDGFRα+/Integrin α2+ Fibroblasts Orchestrate Tumor Budding in Oral Squamous Cell Carcinoma via Mechano‐Metabolic Symbiosis: E‐Cadherin/Integrin α2β1 Adhesion and Mitochondrial Transfer
Source: Adv Sci (Weinh). 2026 Jun 30:e76385. Online ahead of print. doi: 10.1002/advs.76385 (PMC13337076; doi:10.1002/advs.76385)
Supplement: Supplementary file 1 — Supporting File 1: advs76385‐sup‐0001‐SuppMat.docx. [file ADVS-9999-e76385-s002.docx]

**Supplementary Materials**

PDGFRα⁺/integrin α2⁺ Fibroblasts Orchestrate Tumor Budding in Oral Squamous Cell Carcinoma via Mechano-Metabolic Symbiosis:

E-Cadherin/Integrin α2β1 Adhesion and Mitochondrial Transfer

Yufang Liu^1, 2^, Jiao Li^2, 3^, Juan Liu^1, 2^, Qi Dong^1, 2^, Haoyang Zhang^1, 2^, Yanjin Wang^1, 2^, Huibing Li^1, 2^, Ye Guan^1, 2^, Lei Cao^1, 2^, Manqing Zhang^1, 2^, Fangning Guo^1, 2^, Xue Liu^2, 4^, Zhen Yang^5^, Mengmeng Lu^6^, Hui Liu^6^, Laiping Zhong^7^, Tong Ji^8*^, Tingjiao Liu^1, 2^*

Correspondence to: tingjiao_liu@fudan.edu.cn

**This PDF file includes:**

Materials and Methods

Figures S1 to S14

Materials and Methods

**Carcinoma cell Lines.** OSCC cell lines, CAL-27 (RRID: CVCL_1107) and UM-SCC-1 (RRID: CVCL_7707), were purchased from Guangzhou Cellcook Biotech Co., Ltd. The CAL-27 was derived from a tongue lesion in a 56-year-old Caucasian male. UM-SCC-1 was isolated from a tumor on the floor of the mouth of a male patient. The cells were authenticated by Short Tandem Repeat profiling and were confirmed to be free of mycoplasma contamination. CAL-27 cells were cultured in DMEM with high glucose (Hyclone, Logan, UT, USA) and UM-SCC-1 cells were cultured in RPMI 1640 medium (Hyclone, Logan, UT, USA). Both cell lines were maintained in medium supplemented with 10% Fetal bovine serum (FBS), 100 U/mL penicillin, and 100 U/mL streptomycin.

**Primary CAFs Isolation and Culture.** CAFs were isolated from fresh human OSCC tissues obtained from Zhongshan Hospital, Fudan University, China. The use of these clinical samples was approved by the Ethics Committees of Shanghai Stomatological Hospital and Zhongshan Hospital, Fudan University, and conducted in accordance with recognized ethical guidelines. Briefly, sterile fresh OSCC tissues were collected from the surgical specimens and stored in a tissue storage solution (MACS, 130-100-008). The tissues were washed with PBS containing 2% penicillin/streptomycin and cut into small pieces (1 mm³). The tissues were subsequently digested with an enzyme mixture containing 1 mg/mL Collagenase IV (Worthington, LS004188), 2 mg/mL Neutral Protease (Abmole, M10231), and 0.01–2 U/mL Hyaluronidase (Abmole, M9941) for 30 minutes (min), with pipetting every 5 min to ensure complete digestion. Digestion was terminated by adding an equal volume of DMEM/F12 (Hyclone, Logan, UT, USA) supplemented with 10% fetal bovine serum (FBS; ScienCell, Carlsbad, CA, USA), 100 U/mL penicillin, and 100 U/mL streptomycin (Hyclone), and filtered through a 70-μm cell strainer. The filtrate was centrifuged and the pellet was resuspended and seeded into cell culture dishes. The medium was replaced every 2–3 days. Given the differential sensitivity of epithelial cells and CAFs to 0.25% trypsin (Sigma-Aldrich), CAFs were selectively harvested by brief trypsinization (approximately 2 min) under careful observation using a phase-contrast microscope. The remaining cells, predominantly epithelial cells, were collected after trypsinization for 2 min. The CAFs were named CAF-L1/L2/L3/L4/L5. CAFs were cultured in DMEM/F12 (Hyclone, Logan, UT, USA) supplemented with 10% fetal bovine serum (FBS; ScienCell, Carlsbad, CA, USA), 100 μg/mL Primocin™ (Invivogen, ant-pm-05), 100 U/mL penicillin, and 100 U/mL streptomycin (Hyclone).

**Immunofluorescence.** Organoids were fixed in 4% paraformaldehyde (Solarbio, P1110) for 30 min at room temperature and permeabilized with 0.25% Triton X-100 (Sigma) in PBS for 30 min, followed by blocking with 5% BSA (V900933; Sigma-Aldrich) in PBS for 1 h. The samples were then incubated with primary antibodies diluted in PBS) overnight at 4°C. The following primary antibodies were used: anti-E-cadherin (1:500, Abcam, ab40772, RRID: AB_731493), anti-integrin α2 (1:100, Abcam, ab208770, RRID: AB_3720976), anti-integrin β1 (1:500, Abcam, ab30394, RRID: AB_775726), anti-α-catenin (1:200, Invitrogen, PA5-36046, RRID: AB_2553342), anti-β-catenin (1:400, Cell Signaling Technology, 2677, RRID: AB_1030943), anti-YAP (1:50, Santa Cruz, sc-101199, RRID: AB_1131430), anti-P-MLC-2 (1:50, Cell Signaling Technology, 3671, RRID: AB_330248), anti-SNAIL-1 (1:100, Abclone, A5243, RRID: AB_2766076), Actin-Tracker Red-Rhodamine (1:400, Beyotime, C2207S), anti-MT-ATP8 (1:100; ABclonal; A17890, RRID: AB_2861745), anti-LAMB3(1:500; Abcam, ab97765, RRID: AB_10678844), anti-MMP14 (1:50; Absin; abs100043, RRID: AB_3720978), anti-MMP1(1:200; Proteintech; 10371-2-AP, RRID: AB_2297741). After primary antibody incubation, washed three times with PBS and subsequently incubated with Alexa Fluor-conjugated secondary antibodies (all 1:200 dilution; Goat Anti-Mouse IgG H&L Alexa Fluor® 488 [ab150113, RRID: AB_2576208], Alexa Fluor® 594 [ab150116, RRID:AB_2650601], Alexa Fluor® 647 [ab150115, RRID: AB_2687948], Goat Anti-Rabbit IgG H&L Alexa Fluor® 488 [ab150077, RRID: AB_2630356], Alexa Fluor® 594 [ab150080, RRID: AB_2650602]; Abcam, Cambridge, UK) overnight at 4°C in a humidified chamber. The samples were extensively washed with PBS before imaging using a Leica DMi8 confocal microscope (Leica Microsystems, Germany, RRID:SCR_024663).

**Cell Viability Assay.** Viability of the TBO models was assessed on day 5 of culture using a Live/Dead Cell Staining Kit (Beyotime, C2015M). On day 5 of culture, the TBO models were washed thrice with PBS and stained using the Beyotime Live/Dead Cell Staining Kit. The staining procedure was performed as follows. TBO was incubated with a working solution of Calcein AM and propidium iodide (PI) at 37°C for 30 min in the dark. Fluorescence imaging was carried out with a fluorescence microscope using standard filter sets for Calcein AM (Ex/Em = 494/517 nm, green) and PI (Ex/Em = 535/617 nm, red).

**Cell Transfection.** Cells were seeded in 6-well plates and cultured until reaching approximately 70% confluence prior to transduction. For lentivirus-mediated overexpression of hCOX8A-EGFP, CAFs were transduced at a multiplicity of infection (MOI) of 25, whereas OSCC cells were transduced at an MOI of 50. For CRISPR-Cas9-mediated knockout of integrin α2, CAFs were transduced with lentiviral particles at an MOI of 50. All transduction procedures were performed for 24 h, after which the medium was replaced. To eliminate uninfected cells, puromycin was added to the culture medium at a final concentration of 2.5 µg/mL. Knockout efficiency was validated by Western blotting prior to downstream experiments.

**Western Blotting.** Cells were collected, and proteins were extracted using RIPA buffer (Solarbio, R0010) supplemented with phosphatase (MCE, HY-K0021) and protease (MCE, HY-K0010) inhibitors. The total protein content was determined using a Protein Quantification Kit (Abbkine, KTD3001). Total lysates were subjected to SDS-PAGE and transferred onto a Nitrocellulose Blotting Membrane (Merck Millipore, HATF00010), which was then blocked with 5% BSA in Tris-buffered saline with Tween 20. The membrane was immunostained with primary antibodies against PDGFRα (1:500, Abcam, ab203491, RRID:AB_2892065), PDGFA (1:200; Absin, abs119773, RRID: AB_3720980), and GAPDH (CUSABIO, CSB-MA000071M1m, RRID: AB_3720981), followed by incubation with the corresponding HRP-linked secondary antibodies (Thermo Fisher, 31430, RRID:AB_228307.) and visualization using an enhanced chemiluminescence assay (ECL, Thermo Fisher, [A38556](https://www.thermofisher.cn/order/catalog/product/A38556)). All original, uncropped western blot images are provided in the Supplementary Information.

**Transwell in Vitro Cell Migration and Invasion Assays.** For the migration assay, OSCC cells were digested and resuspended in complete medium supplemented with 10% FBS at a density of 2.0×10⁵ cells/mL. The CAFs were resuspended in serum-free medium and adjusted to the same density. OSCC cell suspension (600 μL) was seeded into the lower chamber of a transwell plate, with a control group left without OSCC cells. Then, 200 μL of the CAF suspension was added to the upper chamber inserts (8 μm pore size), along with 2.5 µM Imatinib. The plates were incubated at 37°C and 5% CO2 for 24 h. After incubation, the medium was removed and the cells were washed three times with PBS, fixed with 4% PFA for 20 min, and stained with 0.05% (w/v) crystal violet for 10 min at room temperature. The inserts were then thoroughly rinsed with deionized water to remove the excess dye. Non-migrating cells on the apical side of the membrane were gently removed. Migrated cells on the underside of the membrane were observed and imaged under 10× and 20× magnifications. Cell counts were performed, and statistical analysis was performed using GraphPad Prism 10.1.2(RRID:SCR_002798), with a p-value < 0.05 considered statistically significant. The only difference in the invasion assay was that the Transwell inserts were pre-coated with 100 μL of Matrigel (354234, Corning) diluted 1:5 in cold serum-free medium, polymerized at 37°C for 24 hours, prior to the seeding of CAFs.

**Scanning Electron Microscopy.** CAF were mixed with OSCC cells at a ratio of 1:2 and seeded onto cell culture slides (Biosharp, BS-14-RC). After culturing for 24 h, the cells were washed thrice with PBS. The samples were then fixed with electron microscope fixative (Servicebio, G1102) at room temperature for 2 h, followed by storage at 4°C. The fixed samples were washed thrice with 0.1 M phosphate buffer (PB, pH 7.4) for 15 min each. Subsequently, the samples were fixed with 1% osmium tetroxide (Ted Pella Inc., 18456) in 0.1 M phosphate buffer (PB, pH 7.4) at room temperature in the dark for 1–2 h. The samples were washed again three times with 0.1 M phosphate buffer (PB, pH 7.4) for 15 min each. The tissues were then dehydrated using a graded ethanol series (30, 50, 70, 80, 90, 95, 100, and 100%) for 15 min each, followed by 15 min in isoamyl acetate. The samples were then dried in a critical-point dryer (Quorum, K850). The samples were mounted on conductive carbon double-sided tape and sputter-coated with gold for approximately 30 s in an ion sputter coater (HITACHI, MC1000). The samples were observed and imaged using a scanning electron microscope (HITACHI SU8100).

The microstructural topography of the PDMS stamps was examined using a SEM2000 scanning electron microscope. To prevent charging and ensure conductivity, the samples were mounted on aluminum stubs and sputter-coated with a 15-nm-thick copper layer in a MEDATC KT-Z1650PVD coater. SEM imaging was performed with an accelerating voltage of 15 kV and a working distance of 5 mm, operating in the high-vacuum mode to achieve optimal surface detail.

**Immunohistochemistry.** Paraffin sections were dewaxed and rehydrated using a gradient ethanol series (100% for 2 min, 95% for 2 min, 80% for 2 min, and 70% for 2 min) following initial incubation in xylene at 55°C for 30 min with concurrent preheating of the first dewaxing solution. Endogenous peroxidase activity was quenched by immersion in 3% H₂O₂ for 10 min at room temperature (protected from light), with extended incubation for 15-20 min for high-enzyme-content tissues, and fresh H₂O₂ was prepared 5-10 min before use. Antigen retrieval was performed using a microwave (10 mM citrate buffer, pH 6.0) involving two cycles of 3 min at high power followed by 5 min rest, and a final 7 min at medium-low power with gradual cooling to room temperature. For staining, sections were blocked with 5% normal goat serum (Solarbio, SL038) for 30 min at 37°C, followed by primary antibody incubation overnight at 4°C. After PBS washing, secondary antibodies (Proteintech, PR30009, RRID: AB_2934294) were applied for 1 h at 37°C and visualized using DAB substrate for 1-10 min under microscope. Hematoxylin counterstaining was performed for 30 s, and the sections were dehydrated using gradient ethanol (80% for 2 min, 95% for 2 min, 100% for 5 min×2) and cleared with xylene before mounting with neutral resin. The primary antibodies used were as follows: pan-CK (1:1000, HUABIO, HA601094, RRID: AB_3071816) and PDGFRA (1:1000, Abcam, ab203491, RRID: AB_2892065).

**Multiple Immunofluorescence Staining.** Formalin-fixed paraffin-embedded OSCC tissue sections were subjected to multiplex immunofluorescence staining using the tyramide signal amplification (TSA) method. Staining was performed sequentially for three markers, pan-CK, PDGFRα and Vimentin. Briefly, sections were deparaffinized and rehydrated. Antigen retrieval was performed using EDTA buffer (pH 8.0) in a microwave oven. Endogenous peroxidases were blocked with 3% H₂O₂, and non-specific binding sites were blocked with 3% BSA. The staining cycle for each marker consisted of the following steps: incubation with a primary antibody overnight at 4°C, followed by an appropriate HRP-conjugated secondary antibody for 50 min at room temperature, and finally signal development with a fluorophore-conjugated TSA reagent (570 nm, 520 nm, 620 nm) for 10 min. A subsequent round of antigen retrieval was performed between each marker staining cycle to denature and remove antibody complexes from the previous round. The primary antibodies used and their dilution ratios were as follows: ready-to-use pan-CK (ZSGB-BIO, ZM-0069, RRID: AB_2941997), PDGFRα (1:500, Abcam1, ab203491, RRID: AB_2892065), α-SMA (1:2000, Abcam, ab7817, RRID: AB_262054) and vimentin (1:500, Cell Signaling Technology, 5741, RRID: AB_10695459). After all the markers were sequentially stained, the cell nuclei were counterstained with DAPI. The slides were coverslipped using anti-fade mounting medium. Finally, the stained slides were scanned using a digital slide scanner (PANNORAMIC MIDI, 3DHISTECH) to acquire whole-slide images for subsequent analyses.

**Data Analysis**

**ScRNA-Seq Data Processing** **and Quality Control.** Raw sequencing reads were aligned to the human genome (GRCh38) using Cell Ranger 9.0.1 (Feb 6, 2025). Subsequently, the Seurat R package (version 4.3.0) was used to conduct single-cell analyses. Initial quality control was performed by assessing feature and mitochondrial counts. Cells were excluded if their features or mitochondrial counts exceeded the 99th percentile threshold. Additionally, cells with feature counts or unique molecular identifier (UMI) counts below 200 and 500, respectively, were filtered to ensure sufficient sequencing depth. Genes detected in fewer than three cells were excluded to maintain data reliability. Doublet detection was facilitated by the DoubletFinder tool (version 2.0.3), which helped identify and remove potential doublets from the dataset. Data normalization was performed using Seurat's LogNormalize function to prepare the gene count matrix for downstream analysis. The Seurat workflow was followed for scaling and principal component analysis (PCA). To further explore the data, we employed a Uniform Manifold Approximation and Projection (UMAP) for visualization. To mitigate batch effects across samples, Harmony (version 0.1.1) was integrated into the analysis pipeline. Cells were clustered using the Louvain method, and UMAP was used to visualize the clusters based on principal components that cumulatively contributed to a variance of greater than 85%. Differentially expressed genes across cell clusters were identified using the Wilcoxon rank-sum test (equivalent to the Mann-Whitney U test) in the Seurat package (v4.3.0; RRID:SCR_016341) via its FindAllMarkers function. This non-parametric test is appropriate for ordinal-scale data, such as gene expression values, and requires that the compared cell populations are independent.

**Assignment of Cell Types.** Cell types were identified by marker genes selected from previous studies and the CellMarker database (DOI: 10.1093/nar/gky900), with the highest enriched cell type assigned (*p*< 0.05, Bonferroni correction). Clusters lacking significant enrichment were labeled "Unassigned" and manually annotated using the Loupe Browser (10X Genomics) through UMAP/t-SNE visualization overlays.

**Over-Representation Analysis (ORA) Enrichment Analysis.** The differentially expressed genes identified through our analysis were systematically categorized into their corresponding biological pathways via enrichment analysis. GO and KEGG (RRID:SCR_012773) enrichment analyses were performed using clusterProfiler (v4.6.0), and *p*< 0.05 was set as the threshold.

**Gene set enrichment analysis (GSEA).** To explore the distinct biological states or functions of cells in our study, we employed the R package ClusterProfiler (version 4.6.0) to perform GSEA (RRID:SCR_003199). The hallmark gene sets and pathway background files essential for GSEA were sourced from the Molecular Signatures Database (MSigDB) at https://www.gsea-msigdb.org/gsea/msigdb. The differentially expressed genes were ranked according to their fold-change values in descending order. Subsequently, the GSEA function of ClusterProfiler (RRID:SCR_016884) was applied to the sorted gene list using the default parameters. The resultant enriched pathways were visualized, providing a comprehensive view of the biological processes and functions that were significantly altered in the context of our study.

**Gene set variation analysis (GSVA).** To discern the biological states or functional differences among cells, we utilized the GSVA R package to compute the gene set variation score. The hallmark gene sets and pathway background files were procured from the Molecular Signatures Database (MSigDB) (https://www.gsea-msigdb.org/gsea/msigdb). The analysis was performed using the default parameters of the GSVA package. The outcome of this process was then translated into visual representations, including a bar plot that illustrates the differences in pathway activity and a heatmap depicting the gene set variations across the samples.

**scRNA velocity analysis.** RNA velocity analysis is a pivotal tool for validating the developmental trajectories of cells. To perform this analysis, we used the Python package 'Scvelo' (RRID:SCR_018168). The initial step involved converting the input data into a loom format that is compatible with the Scvelo package. Subsequently, we employed the 'filter_and_normalize' function to select the top 2000 genes based on the count of unspliced and spliced transcripts, ensuring a robust representation of RNA velocity. The first and second moments, which are essential for estimating the velocity of each gene, were then computed. The RNA velocities were calculated using the 'stochastic' function, providing a measure of the rate of change in gene expression over time. Following the computation of velocities, we proceeded with dimensionality reduction using t-SNE or UMAP to facilitate the visualization. Finally, downstream visualization of the RNA velocity analysis was accomplished with the 'scv.pl.velocity_embedding_grid' and 'scv.pl.heatmap' functions, offering a clear depiction of the cells' developmental trajectories and their velocities.

**Inferring cell-cell communication with cellphoneDB.** For a comprehensive examination of cell-cell interactions, we deployed CellPhoneDB (version 5.0) by Vento-Tormo et al. (2018), a robust statistical tool designed for the prediction of cell-cell interaction networks. Our analysis focused on genes that were expressed in at least 0.1% of the cells within any given cell type, thereby establishing a threshold for gene inclusion. To bolster confidence in our interaction predictions, we conducted pairwise subtype analysis. This involved a rigorous permutation test, where the labels of all cells were randomly shuffled 1000 times. This process allowed us to ascertain the statistical significance of each ligand-receptor pair interaction between the different cell subtypes. Interactions with a p-value of less than 0.05 were deemed significant and were subsequently selected for further consideration. The visualization of these significant interactions was facilitated by the built-in functions of CellPhoneDB: the 'dot_plot' function was utilized to create a bubble diagram, while the 'heatmap_plot' function generated a circle diagram. These visual representations effectively convey the nature and extent of the interactions between various cell types.

**IsRNA-Seq Data Processing.** Images were acquired using a 3D-Hitech Pannoramic® MIDI II with a pco.edge 4.2bi camera and a 20x objective lens. A round of DAPI images was selected as the reference image, and the SIFI algorithm was used to obtain the features of different rounds of DAPI images. The feature points of different rounds of DAPI images were matched, and the obtained matching feature points were used for affine transformation to register the images of different rounds. Then, the TopHat (RRID:SCR_013035) of OpenCV (RRID:SCR_015526) was used for background filtering. The spatial localization and quantification of RNA were confirmed by recognizing the real signal and decoding it.

**TCGA analysis.** The mRNA expression profiles and corresponding clinical information of head and neck squamous cell carcinoma (HNSCC) patients were downloaded from The Cancer Genome Atlas (TCGA) database. The expression levels of PDGFRα were extracted from the mRNA expression profiles, and the median PDGFRα expression value across all samples was used as the cut-off point to classify samples into high and low PDGFRα expression groups. The CAF score was calculated using a previously described method. Briefly, 11 overall survival (OS)-related CAF hub genes were identified using univariate Cox regression analysis (*p* < 0.05), and these genes were then subjected to least absolute shrinkage and selection operator (LASSO) regression analysis to obtain non-zero regression coefficients. The CAF score for each sample was calculated as the sum of the products of the mRNA expression levels for each gene and their corresponding coefficients. The optimal cutoff value for the CAF score was determined using the “Survminer” R package (RRID:SCR_021094), and the samples were divided into high and low CAF score groups based on this cutoff. According to PDGFRα expression levels and CAF scores, samples were divided into four groups: high expression of PDGFRα with low CAF score, high PDGFRα expression with high CAF score, low PDGFRα expression with high CAF score, and low PDGFRα expression with low CAF score. Differences in overall survival between groups were evaluated by plotting Kaplan-Meier curves and applying the log-rank test using the ‘survival’ package in R (RRID:SCR_021137). Univariate and multivariate Cox regression analyses were performed to evaluate the prognostic value of PDGFRα expression and CAF score.

**Supplementary Figures**


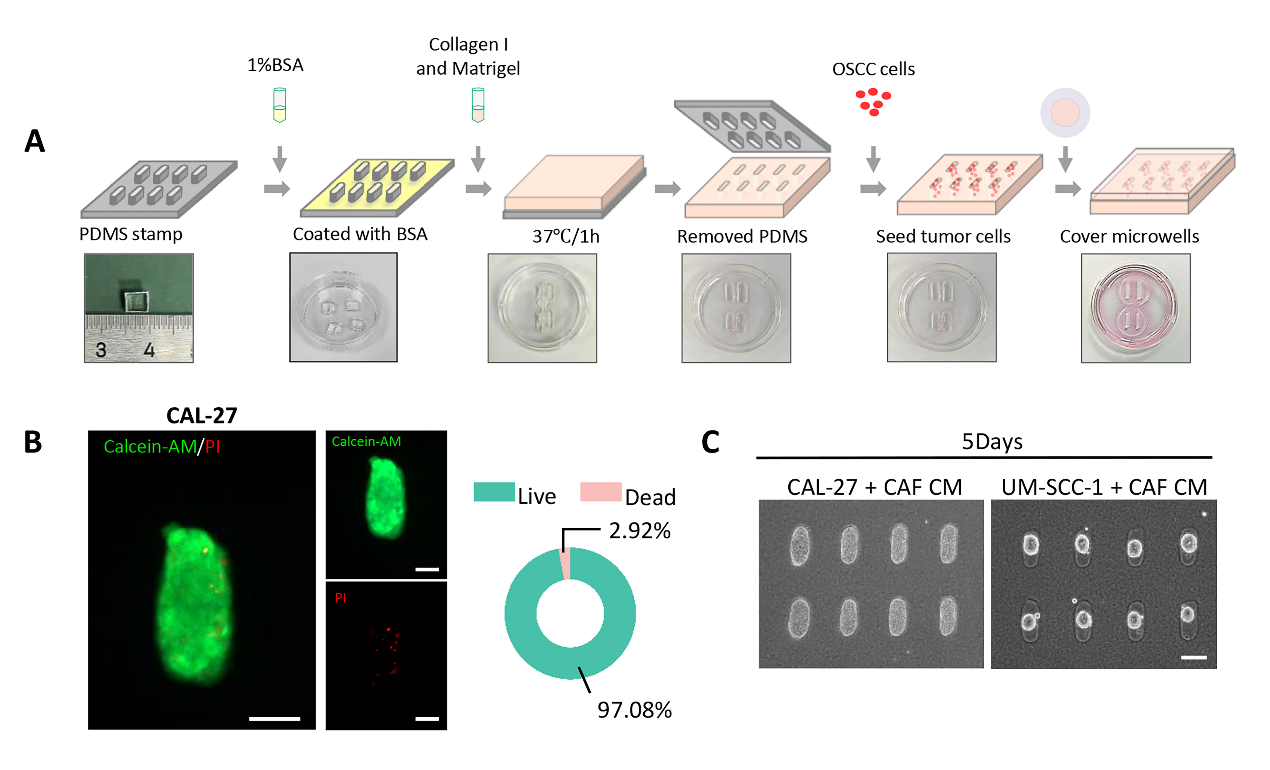


**Figure S1. Schematic diagram and characterization of TPO model.**

(A) Schematic of the establishment of tumor pushing invasion organoids (TPO) and corresponding representative images.

(B) Live/Dead staining of CAL-27 cells in the TPO models, with quantitative analyses (Scale bars = 50 µm).

(C) Representative image of OSCC cells after 5-day induction with CAF-CM, showing no invasion into the surrounding area in the microwells (Scale bars = 100 µm).


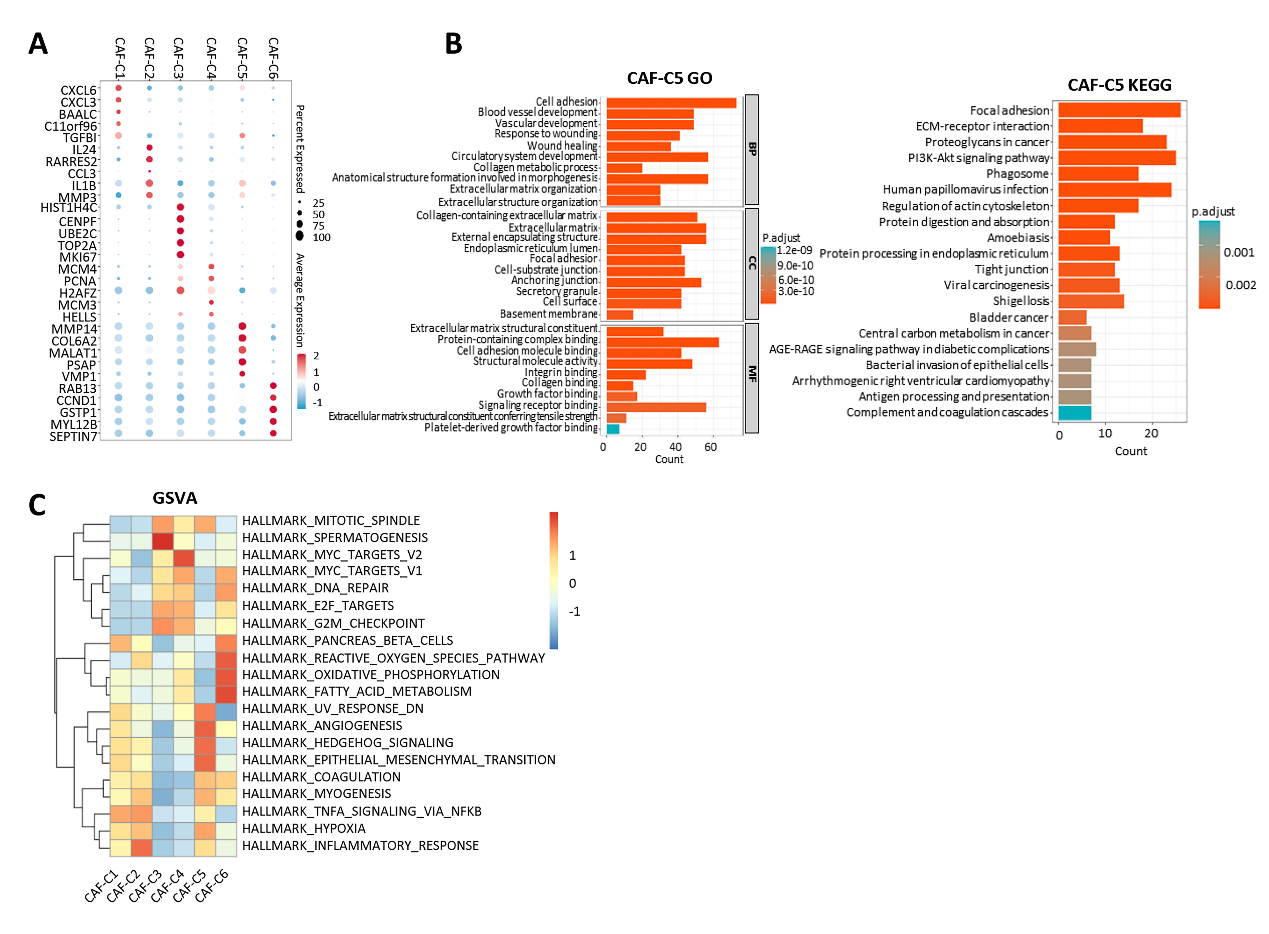


**Figure S2. Single-cell transcriptomic characterizes different CAF subsets.**

(A) Dot plot showing expression of marker genes for different cell clusters.

(B) GO and KEGG enrichment analysis of CAF-C5.

(C) Gene set variation analysis of CAF subtypes.


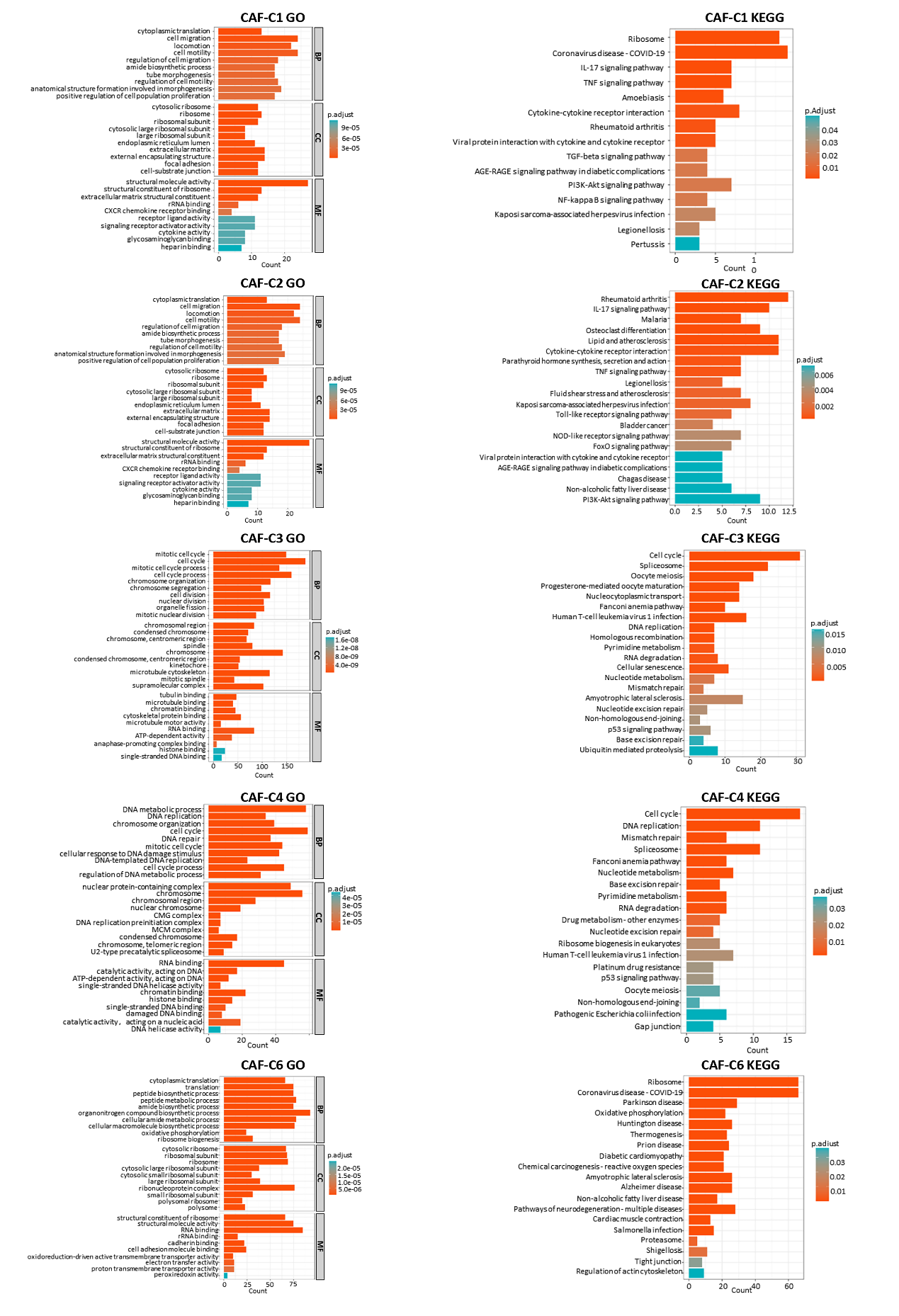


**Figure S3. GO enrichment and KEGG analyses of different CAF subsets.**


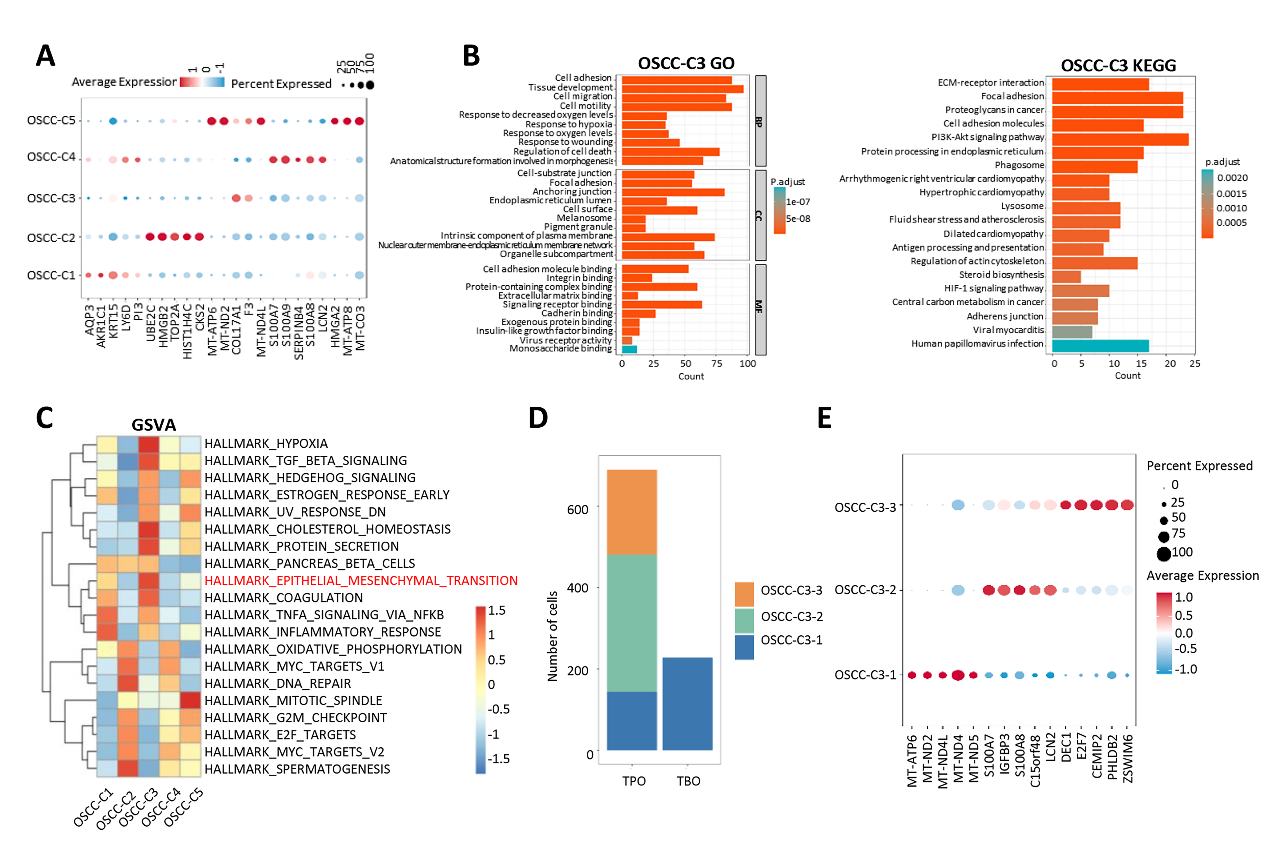


**Figure S4. Identification and functional profiling of OSCC subsets by single-cell transcriptomics.**

(A) Dot plot showing expression of marker genes for different OSCC clusters.

(B) GO enrichment and KEGG analyses of OSCC-C3.

(C) Gene set variation analysis of OSCC subtypes.

(D) Bar plot displaying the re-clustering of OSCC-C3 in scRNA-seq of organoids (TBO and TPO).

(E) Dot plot showing expression of marker genes for OSCC clusters C3-1, C3-2 and C3-3.

**
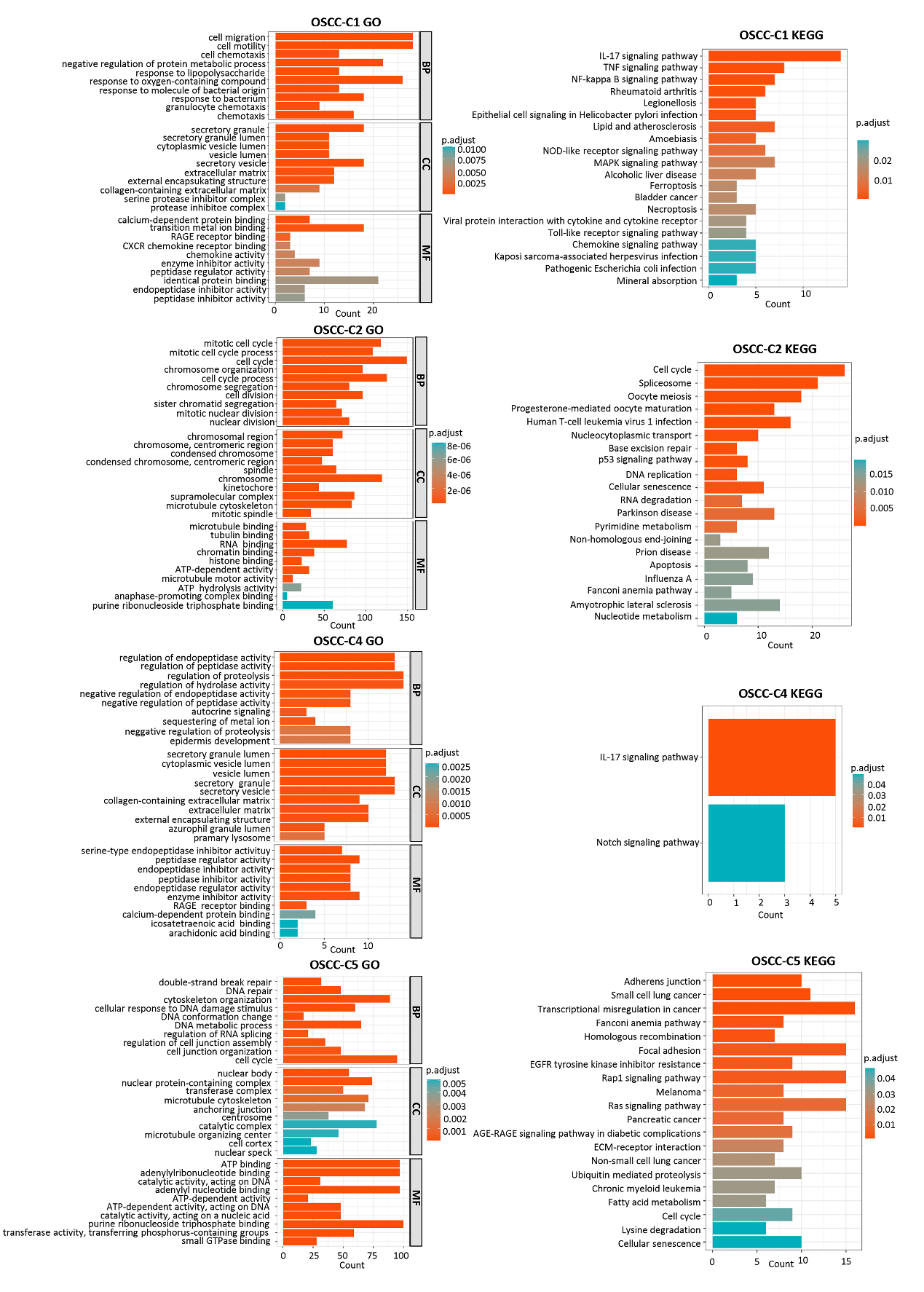
**

**Figure S5. GO enrichment and KEGG analyses of different OSCC subsets.**


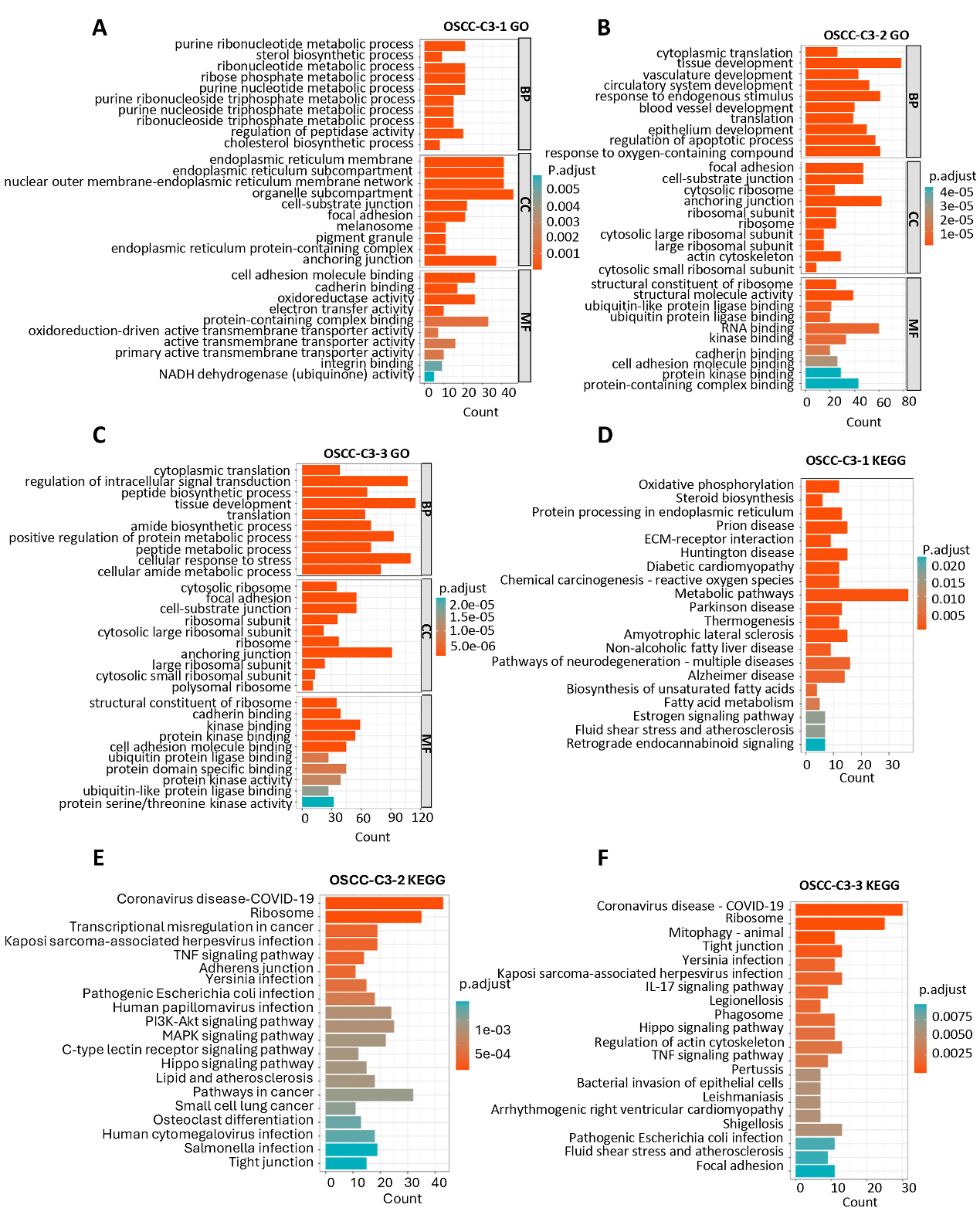


**Figure S6. Single-cell transcriptomic characterizes different OSCC-C3 subsets.**

(A-C) GO enrichment analyses of OSCC-C3 clusters. (D-F) KEGG enrichment analyses of OSCC-C3 clusters


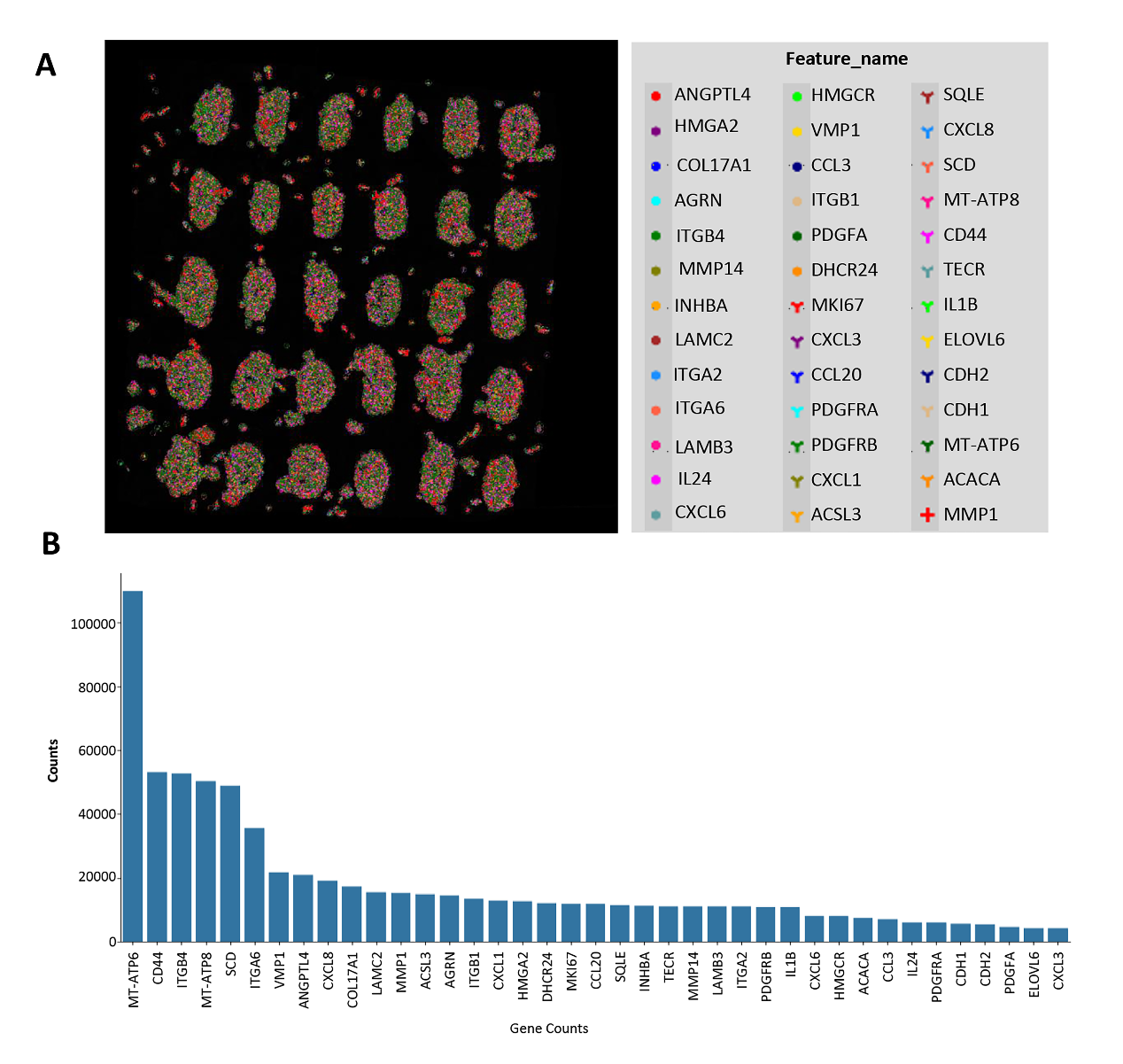


**Figure S7. Spatial RNA sequencing reveals the spatial distribution and expression levels of marker genes in CAF-C5 and OSCC-C3-1.**

(A) Expression patterns of 39 highly expressed genes of OSCC-C3 and CAF-C5 in TBO detected using isRNA-Seq.

(B) Gene counts of 39 highly expressed genes of OSCC-C3 and CAF-C5 in TBO detected using isRNA-Seq.


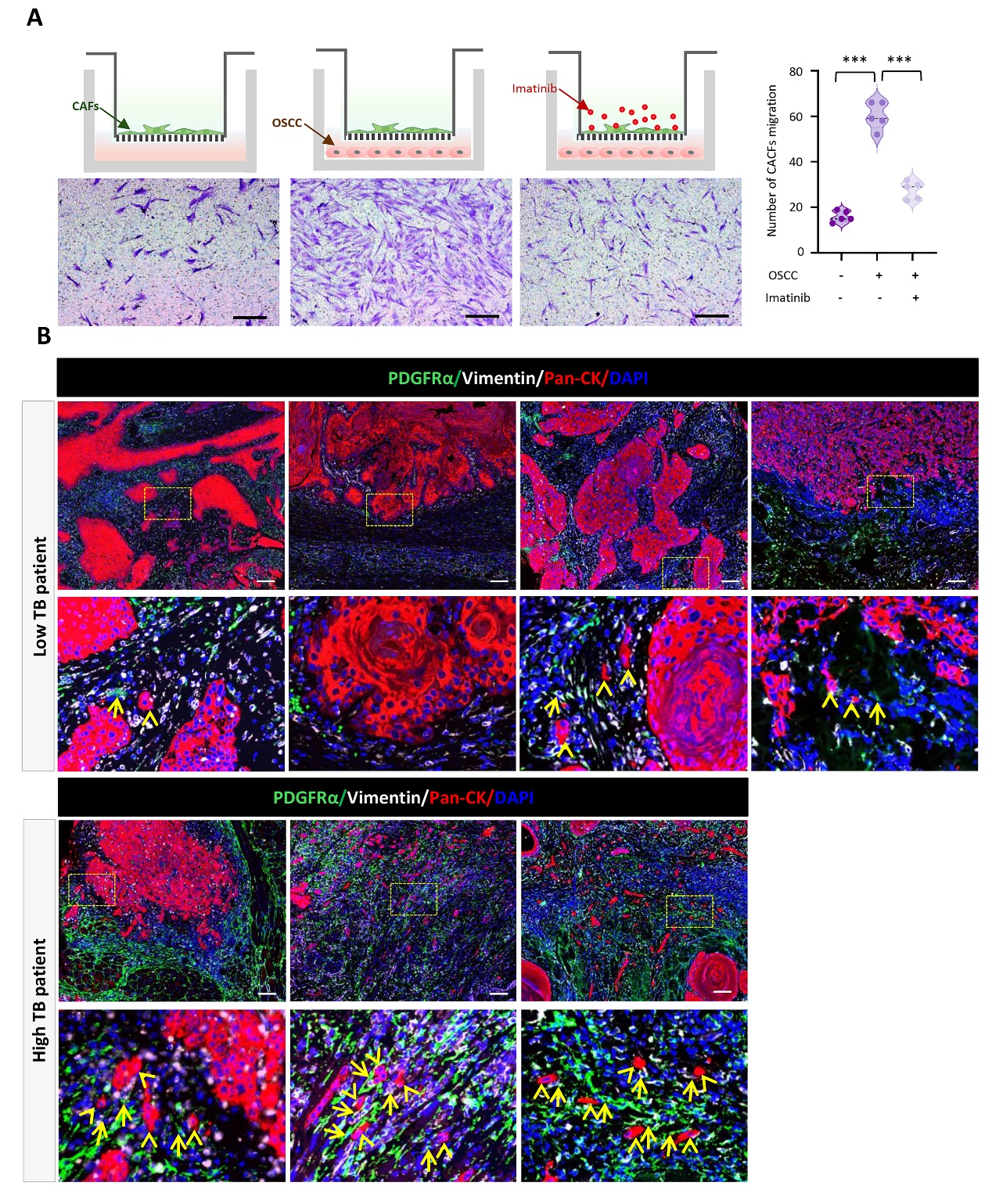


**Figure S8. PDGFRα^+^ CAFs are the key cell subpopulation that induces TB.**

(A) Representative images and quantification of the migration of CAFs with or without OSCC stimulation and inhibitors (imatinib) (Scale bars = 100 µm). Data represent the mean (± SD); n = 5 per group. n = 5 per group. ****p* < 0.001 by One-way ANOVA.

(B) Analysis of PDGFRα⁺ CAFs and TB based on fluorescence imaging, showing their spatial relationship. PDGFRα⁺ CAFs (yellow arrows) are seen surrounding and contacting TB cells (yellow arrowheads) (Scale bars = 100 µm).


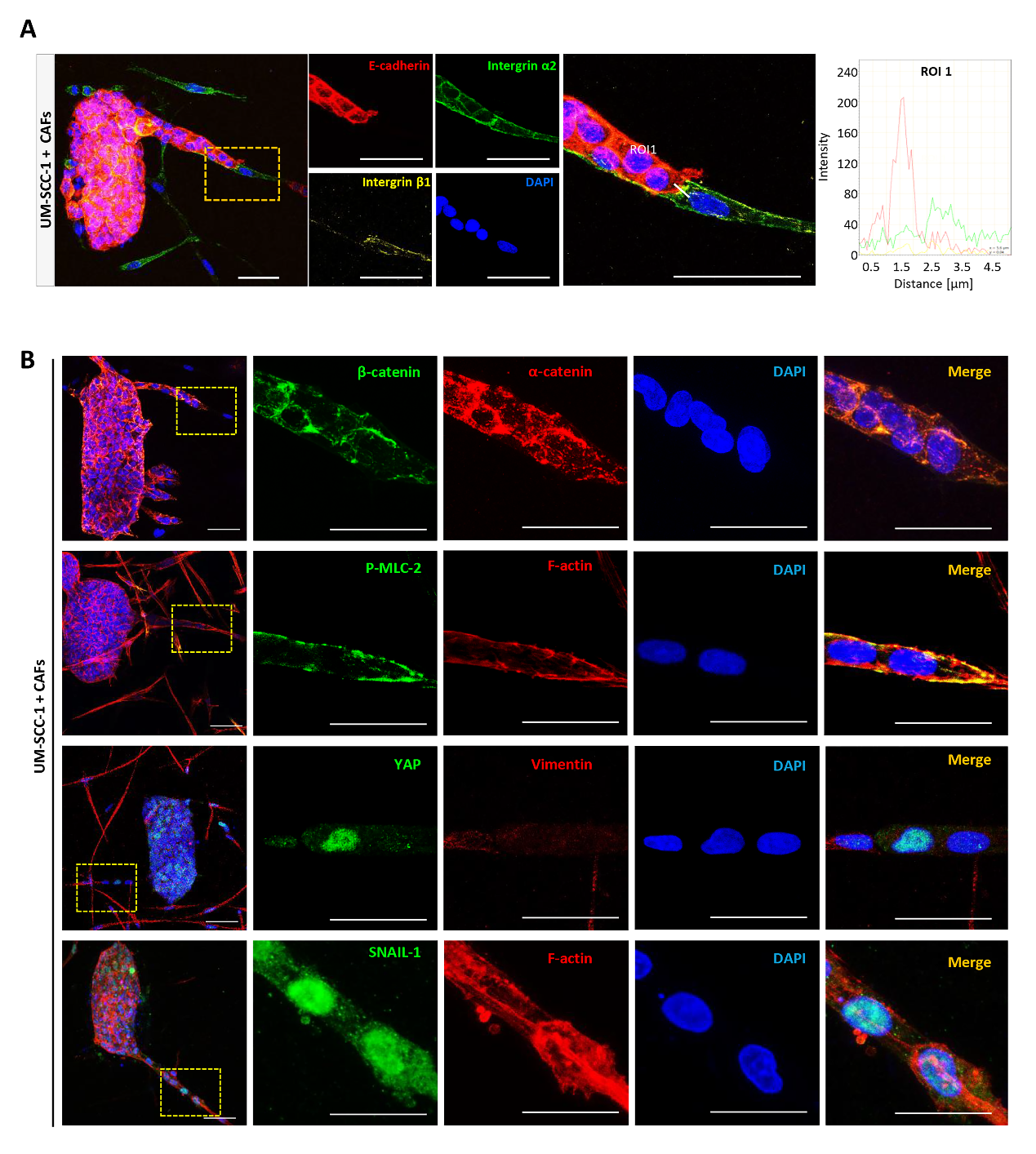


**Figure S9. CAFs activated YAP signaling pathway of OSCC.**

(A) Representative fluorescence images showing co-localization of E-cadherin (OSCC) with integrin α2β1 (CAFs) in the TBO models (Scale bars = 50 µm).

(B) Fluorescence images show the UM-SCC-1 intracellular activated YAP signaling pathway in TBO (Scale bars = 50 µm).


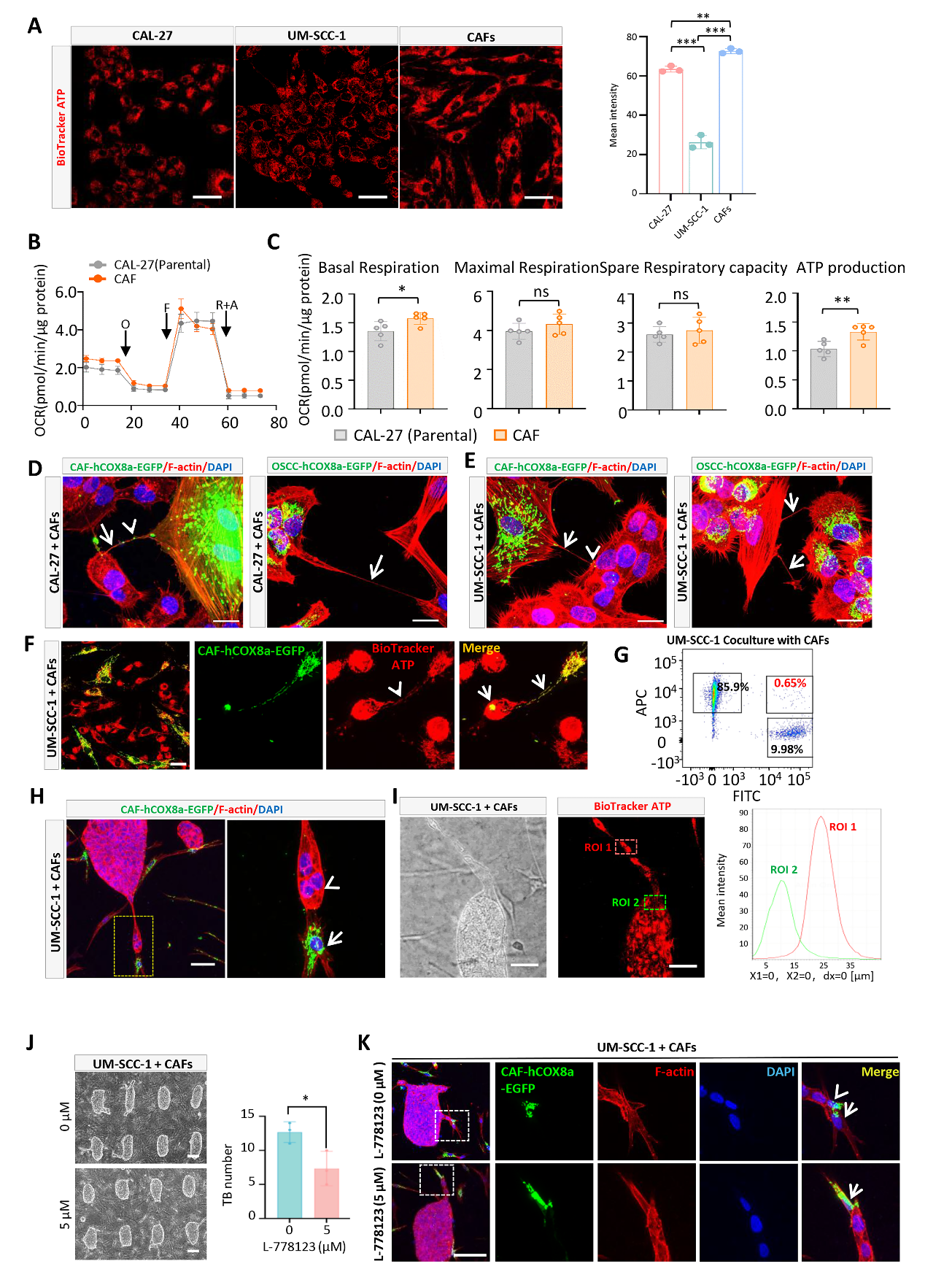


**Figure S10. CAFs transfer mitochondria to OSCC cells via TNTs.**

(A) BioTracker ATP Red staining and quantification in OSCC cells and CAFs (Scale bars = 50 µm). Statistical significance was determined by One-way ANOVA (***p* < 0.01; ****p* < 0.001).

(B) The OCR was recorded in CAL-27 cells and CAF under basal conditions and following the sequential injection of oligomycin (O), FCCP (F), and a mix of rotenone & antimycin A (R+A). Arrows indicate the time points of injections.

(C) The basal respiration and ATP production were significantly increased in the recipient CAL-27 cells and CAF. Data are presented as mean ± SD (n=5 technical replicates per group). Statistical significance was determined by an unpaired two-tailed t-test (ns, not significant and ***p* < 0.01).

(D-E) Representative confocal images of hCOX8a-transfected CAFs co-cultured with OSCC Cells. The white arrow indicates the TNT and the short white arrow points to the mitochondria (Scale bars = 20 µm). Representative confocal images of hCOX8a-transfected OSCC cells co-cultured with CAFs. The white arrow indicates the TNT (Scale bars = 20 µm).

(F) Representative confocal micrographs of hCOX8a-transfected CAFs co-cultured with UM-SCC-1 cells for 24 hours and stained with BioTracker ATP Red. White arrows indicated mitochondria, and short white arrows pointed to the ATP signal produced by these mitochondria (scale bars = 20 µm).

(G) Flow cytometry analysis of E-cadherin+ (APC) hCOX8a+ (FITC) cells after 48-hour co-culture of hCOX8a-transfected CAFs with UM-SCC-1 cells. This population represents UM-SCC-1 cells that have acquired mitochondria from CAFs.

(H) Representative images of hCOX8a-EGFP-CAFs co-cultured with UM-SCC-1 cells in the TBO models showing CAFs delivering mitochondria (green) to UM-SCC-1 cells. Arrow indicated a CAF and the arrow head indicated an UM-SCC-1 cell (scale bars = 50 µm).

(I) Representative brightfield and BioTracker ATP staining images of the TBO models (scale bars = 50 µm). The mean intensity of ROI1 is greater than ROI2.

(J-K) Representative bright-field/immunofluorescent images of CAF-promoted UM-SCC-1 budding with or without L-778123 (scale bars = 100 µm) and quantification of tumor buds. Data are mean ± SD (n = 3 per group); **p* < 0.05 by unpaired t-tests.


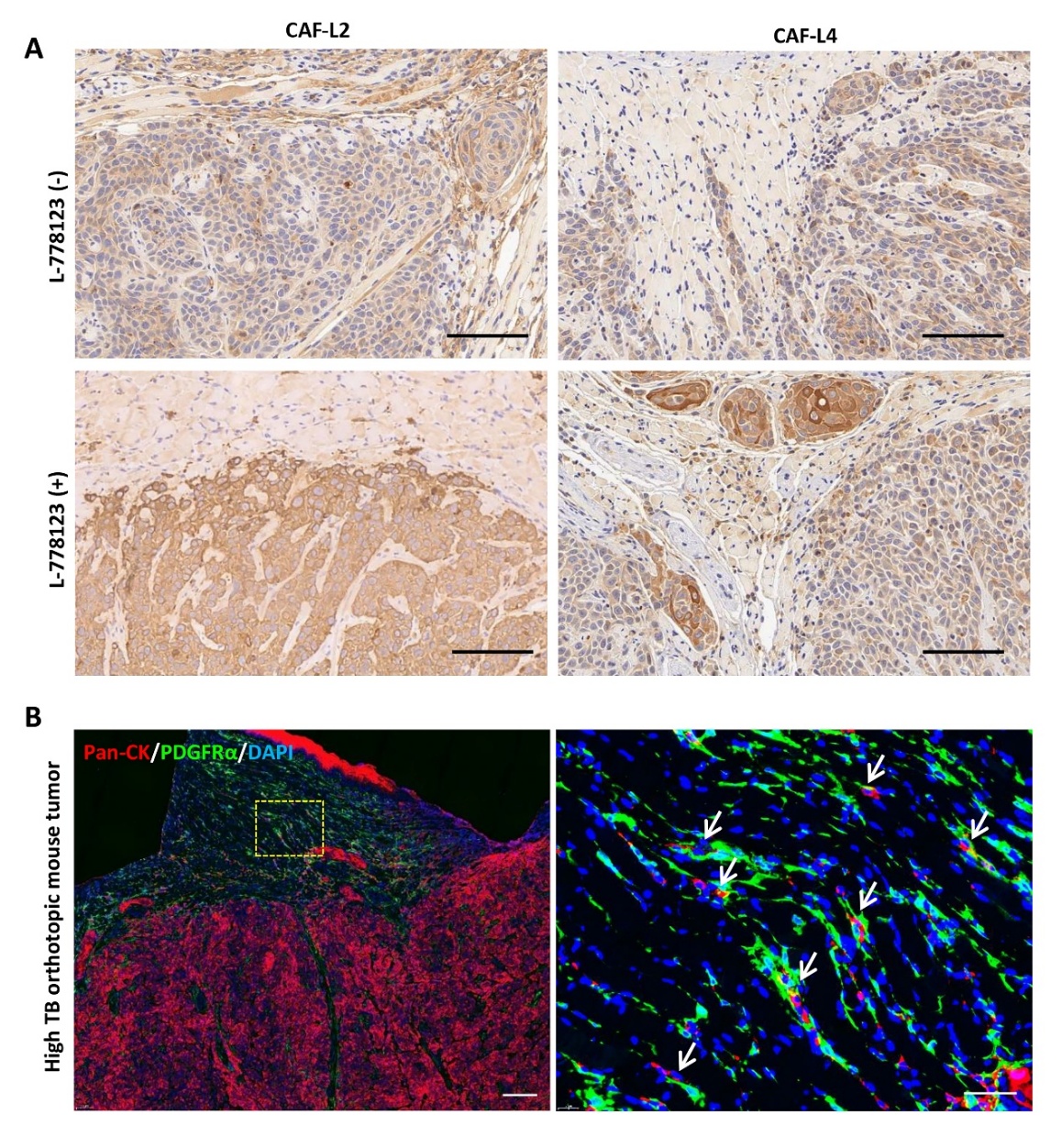


**Figure S11. CAFs induce TB formation in an orthotopic mouse tumor model.**

(A) Immunohistochemical staining of representative tumors (CAF-L2, CAF-L2 + L-778123, CAF-L4, CAF-L4+L-778123) for pan-CK (Scale bars = 100 µm).

(B) Representative fluorescence images show PDGFRα^+^ CAF infiltration in orthotopic OSCC tumors (Scale bar = 200 µm). PDGFRα^+^ CAFs are observed surrounding tumor buds (white arrows) (Scale bars = 50 µm).


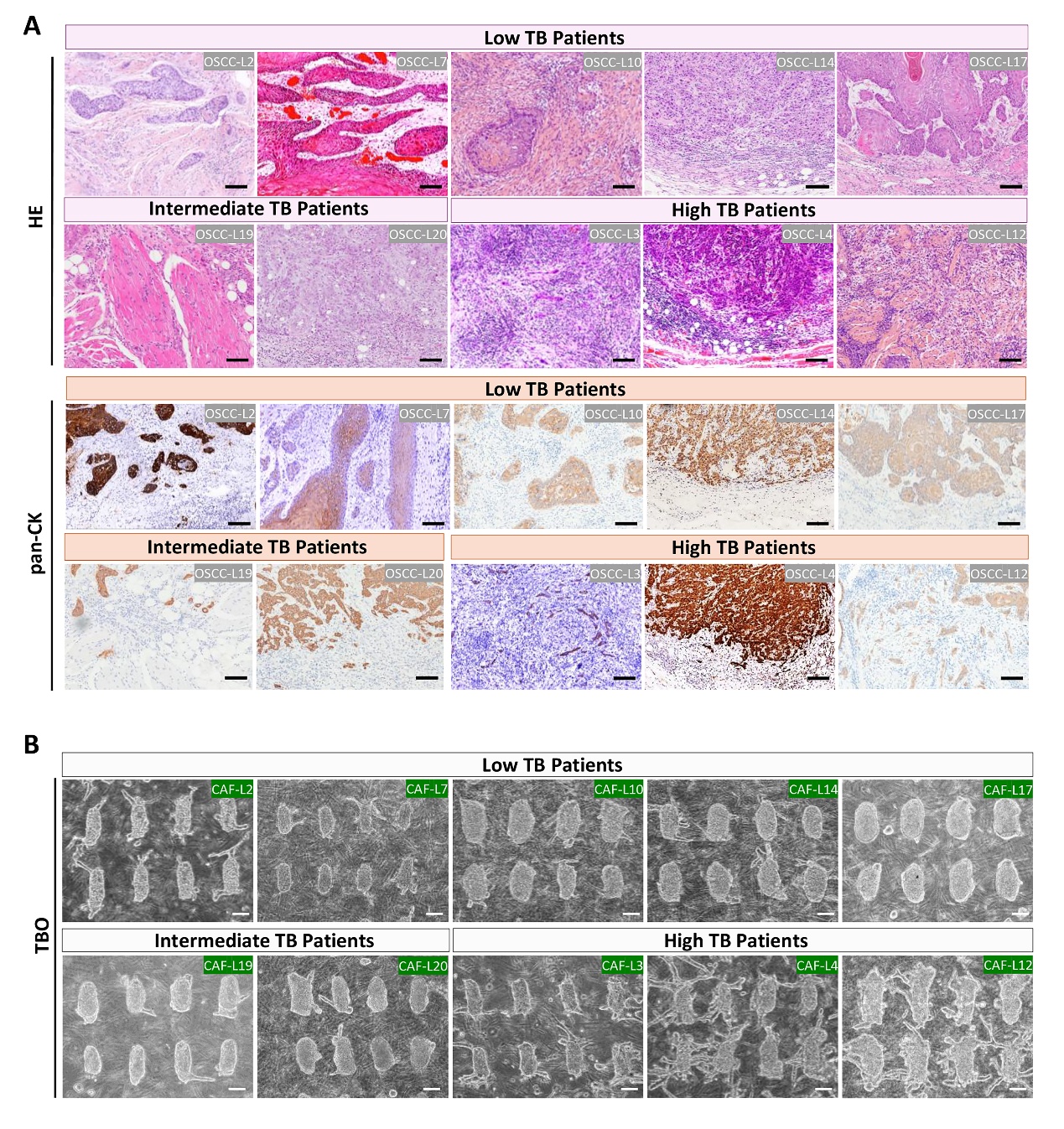


**Figure S12. Analysis of clinical patient samples.**

(A) Representative Heand IHC images of pathological sections from patients with high, intermediate TB and low TB (Scale bars = 100 µm).

(B) Representative images of the TBO models established by CAFs derived from high-, intermediate and low-TB patients, respectively (Scale bars = 100 µm).


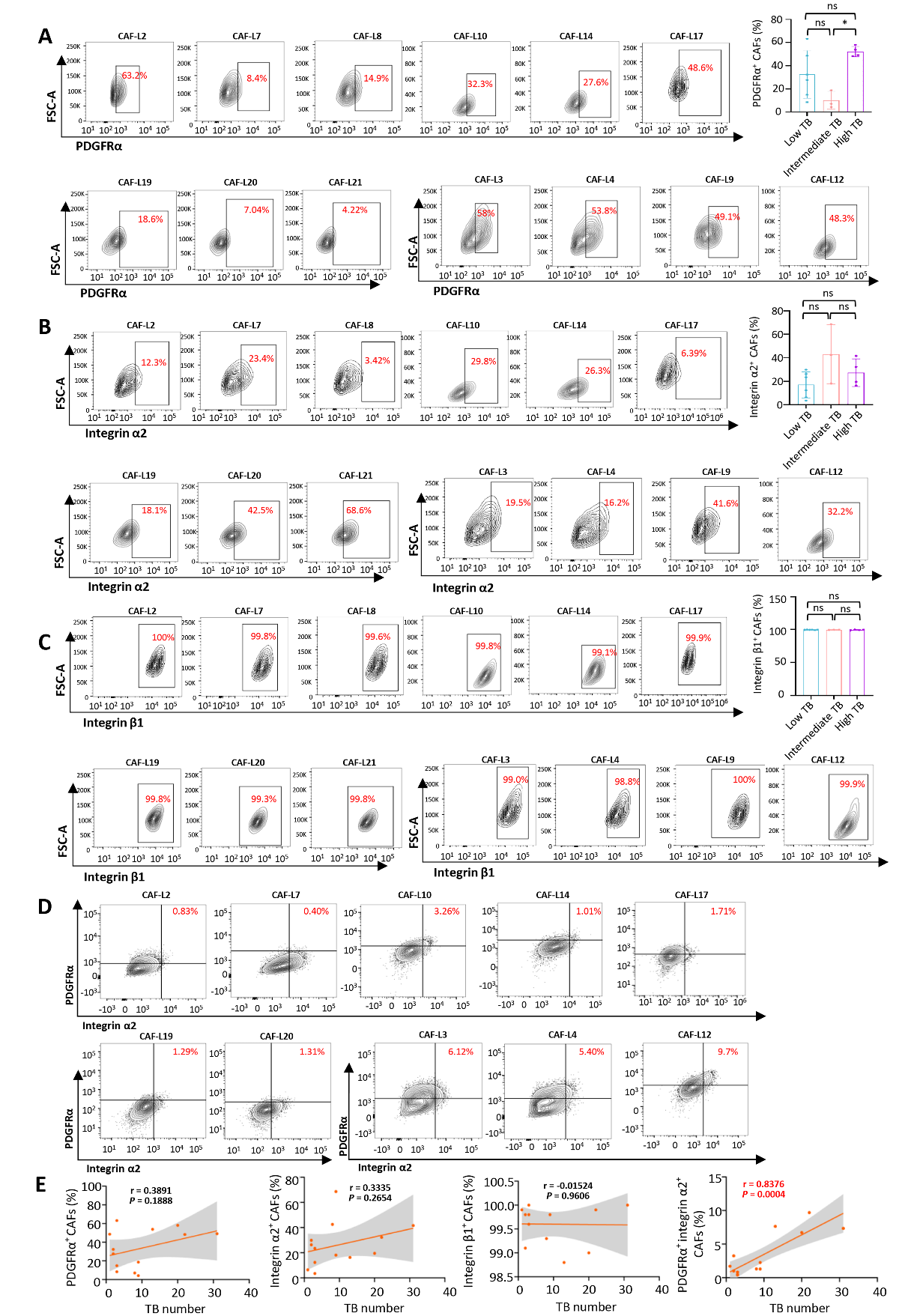


**Figure S13. Flow cytometric and correlation analysis of patient samples.**

(A-D) Flow cytometric analysis of patient-derived primary CAFs showing the proportions of cells positive for PDGFRα, integrin α2, or integrin β1, as well as the percentage of PDGFRα⁺ integrin α2⁺ double-positive cells.

(E) Correlation analysis of the proportions of PDGFRα⁺ CAFs, integrin α2⁺ CAFs, integrin β1⁺ CAFs, and PDGFRα⁺ integrin α2⁺ double‑positive CAFs with TB.

**
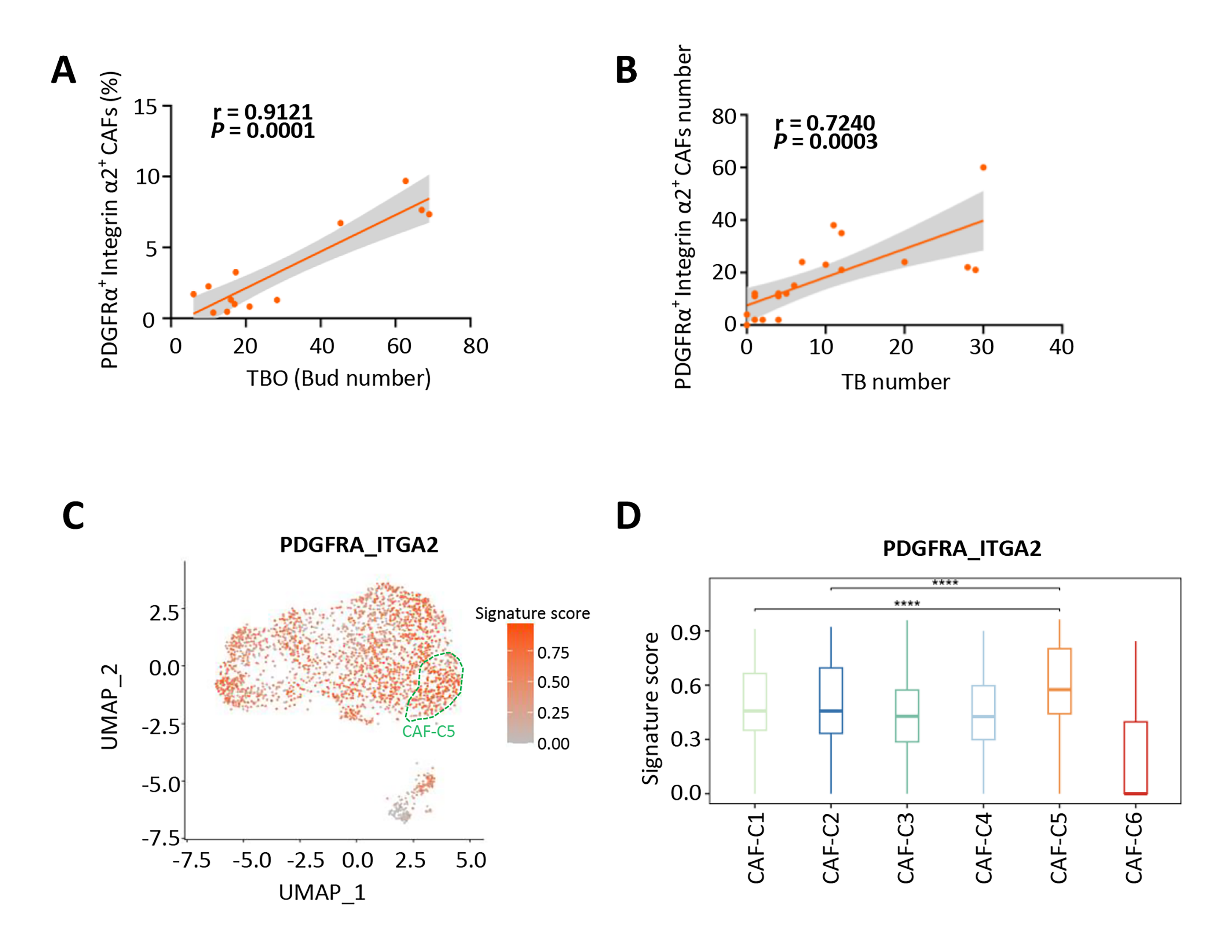
**

**Figure S14. PDGFRα⁺ integrin α2⁺ CAFs are the key CAF subset driving OSCC budding invasion.**

(A-B) Correlation of PDGFRα⁺ integrin α2⁺ CAF percentage with TBO bud number, and correlation of PDGFRα⁺ integrin α2⁺ CAF counts in clinical sections with patient TB number.

(C-D) UMAP and box plots showing the expression scores of PDGFRα⁺/integrin α2⁺ CAFs across different subclusters.
