# Supplementary material for: PDGFRα+/Integrin α2+ Fibroblasts Orchestrate Tumor Budding in Oral Squamous Cell Carcinoma via Mechano‐Metabolic Symbiosis: E‐Cadherin/Integrin α2β1 Adhesion and Mitochondrial Transfer
Source: Adv Sci (Weinh). 2026 Jun 30:e76385. Online ahead of print. doi: 10.1002/advs.76385 (PMC13337076; doi:10.1002/advs.76385)

**Original uncropped Western blot images presented in this study.**

**36 kDa**


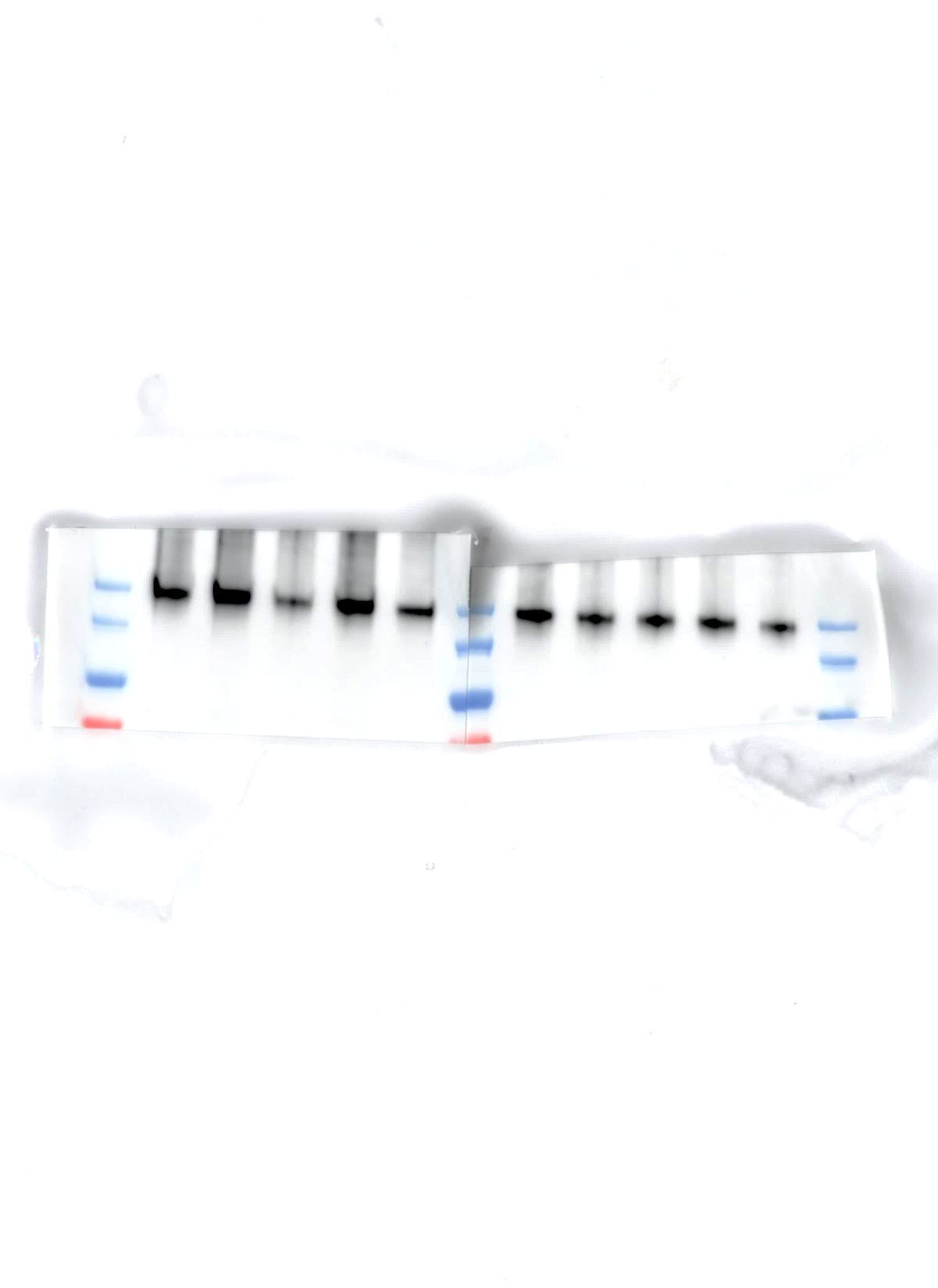


**Integrin α2**

**WT**

**GAPDH**

**NC**

**sgRNA-1**

**36 kDa**

**250 kDa**

**sgRNA-2**

**sgRNA-3**


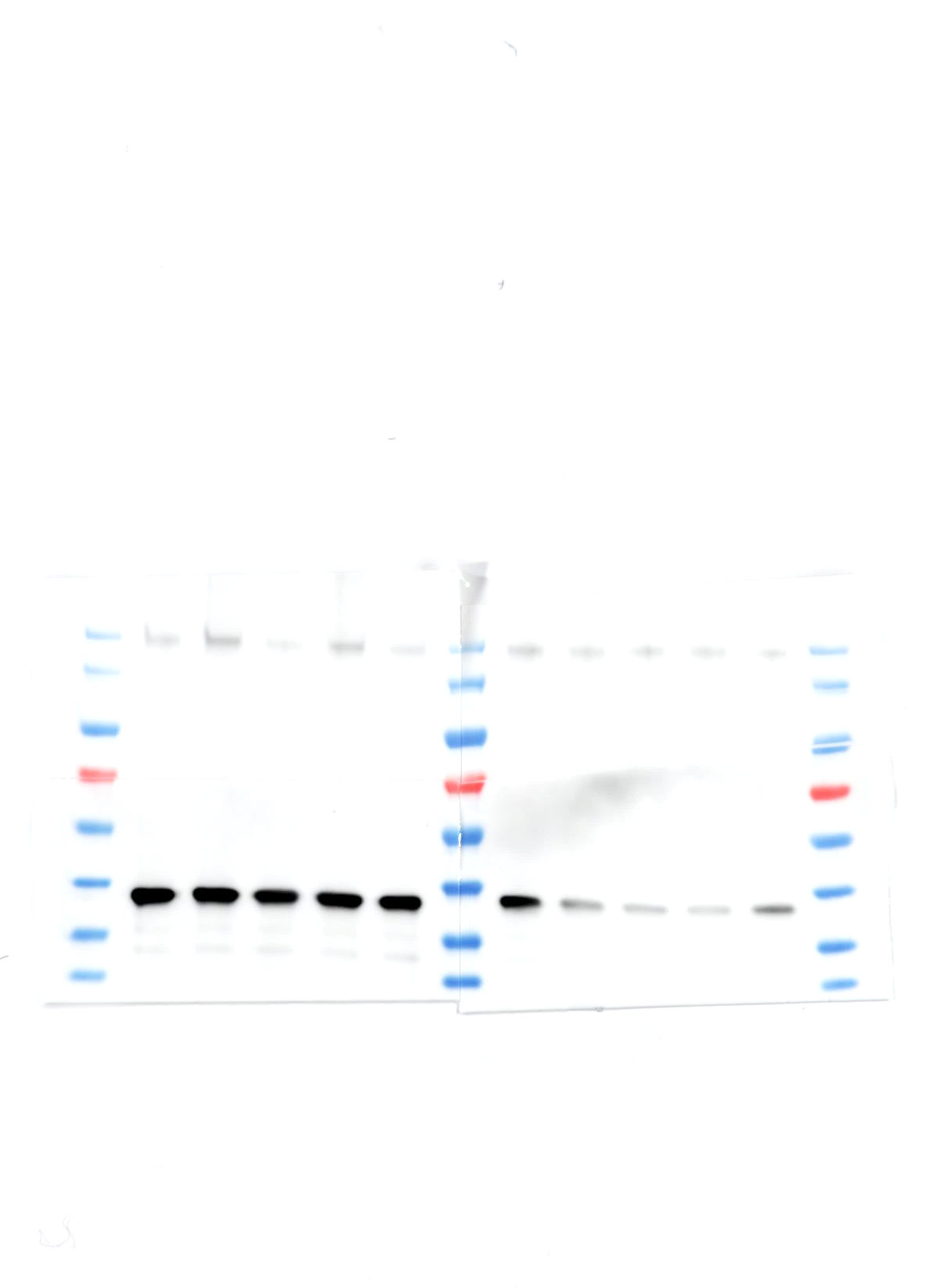


**150 kDa**

**14 kDa**

**CAFs-2**

**CAL-27**

**UM-SCC-1**

**CAFs-1**

**150kDa**

**14kDa**

**PDGFRα**

**PDGFA**

**GAPDH**

**36kDa**


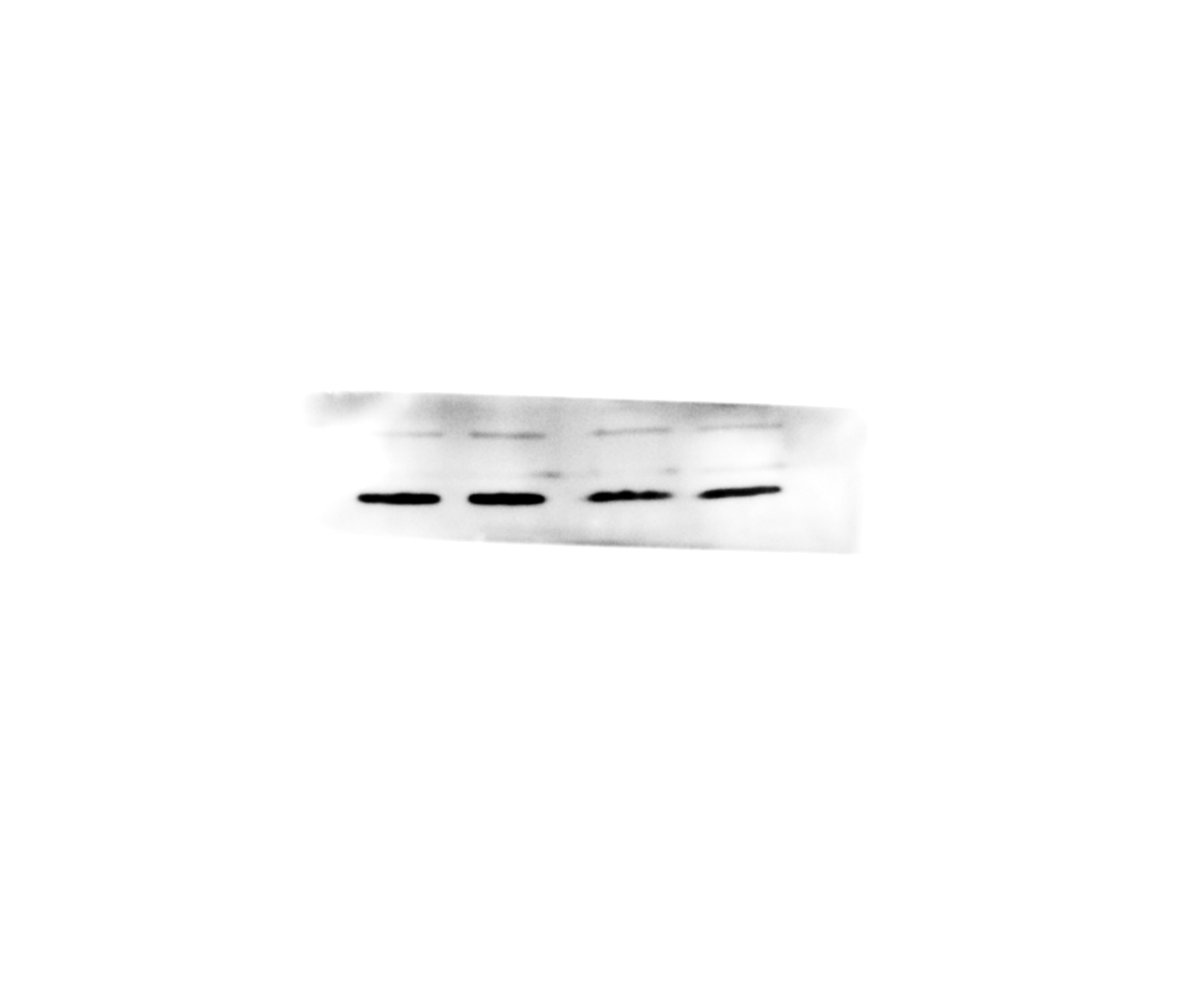

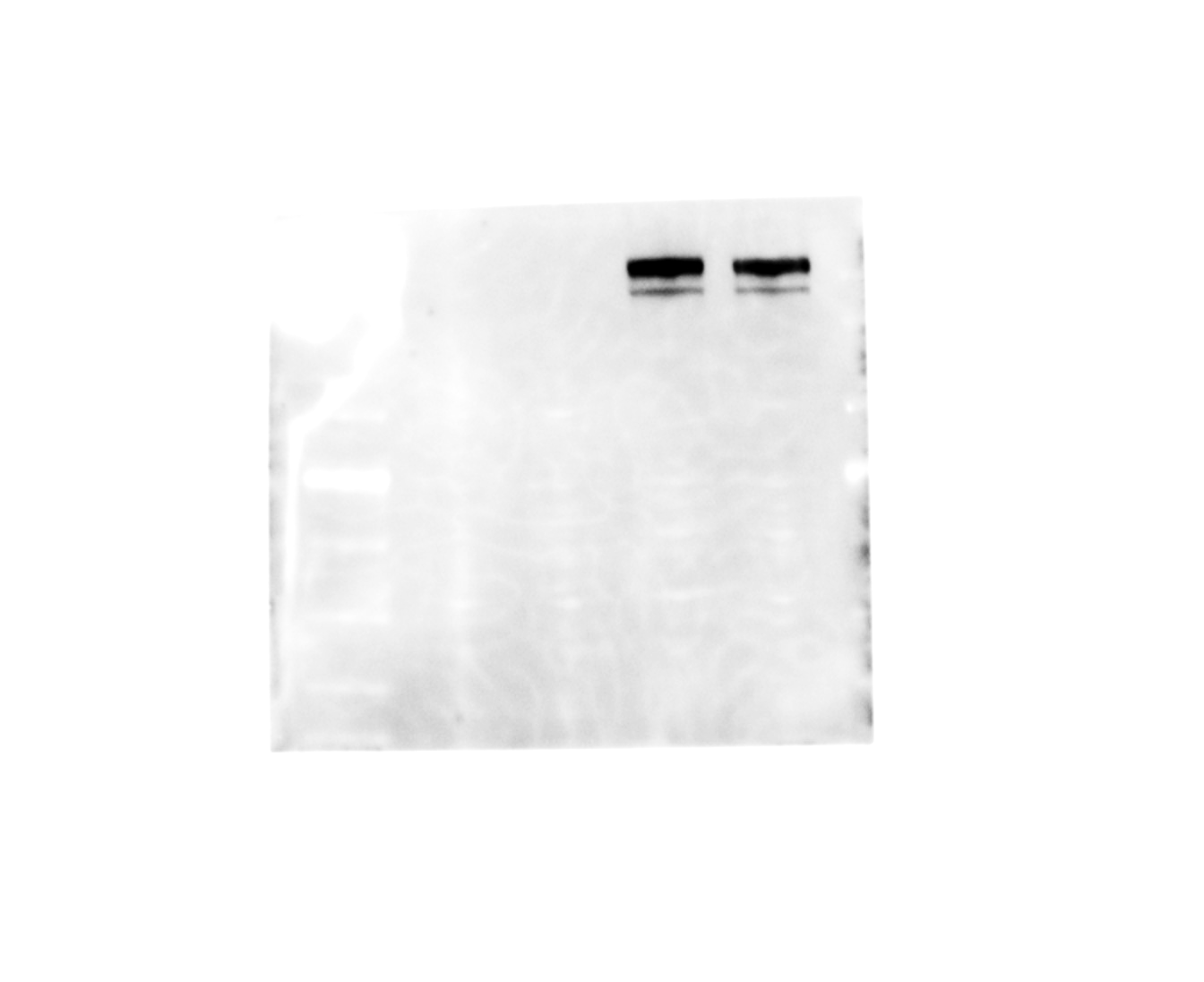

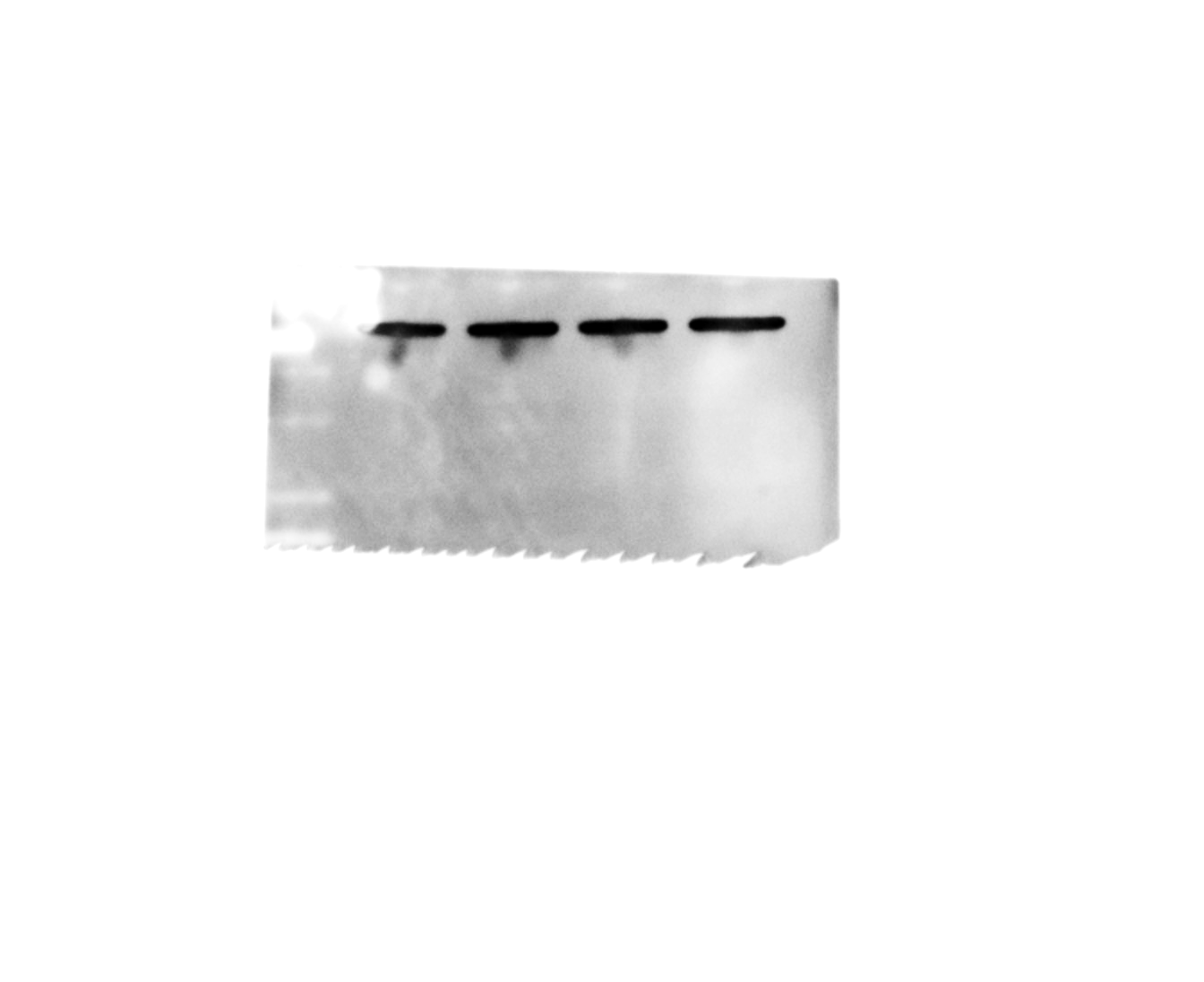

Supplement: Supplementary file 2 — Supporting File 2: advs76385‐sup‐0002‐DataFile.docx. [file ADVS-9999-e76385-s005.docx]
